# Association of Hospital Adoption of Probiotics With Outcomes Among Neonates With Very Low Birth Weight

**DOI:** 10.1001/jamahealthforum.2023.0960

**Published:** 2023-05-12

**Authors:** Leila Agha, Douglas Staiger, Christopher Brown, Roger F. Soll, Jeffrey D. Horbar, Erika M. Edwards

**Affiliations:** 1 Department of Health Care Policy, Harvard Medical School, Boston, Massachusetts; 2 Department of Economics, Dartmouth College, Hanover, New Hampshire; 3 AEA Investors, New York, New York; 4 Vermont Oxford Network, Burlington, Vermont; 5 Department of Pediatrics, The Robert Larner, M.D. College of Medicine, University of Vermont, Burlington; 6 Department of Mathematics and Statistics, College of Engineering and Mathematical Sciences, University of Vermont, Burlington

## Abstract

**Importance:**

For neonates with very low birth weight (VLBW), randomized clinical trials (RCTs) indicate that probiotic treatment decreases the risk of necrotizing enterocolitis (NEC), with smaller decreases in the risk of sepsis and death. There is little evidence on the rate of probiotic adoption in US neonatal intensive care units (NICUs) and whether the benefits seen in trials have materialized in practice.

**Objective:**

To estimate changes in probiotic use among neonates with VLBW and to test whether neonates with VLBW treated at NICUs adopting routine probiotic use experience better outcomes compared with neonates treated at nonadopting NICUs.

**Design, Setting, and Participants:**

This cohort study used Vermont Oxford Network data on neonates with VLBW in US NICUs from January 1, 2012, to December 31, 2019. Data were analyzed from January 2022 through February 2023.

**Exposure:**

Probiotics adoption vs nonadoption. Adopting NICUs were defined as those that currently or previously treated at least 20% of neonates with VLBW with probiotics.

**Main Outcomes:**

The primary outcomes were rates of NEC, in-hospital mortality, and sepsis, defined as bacterial or fungal infection occurring after day 3 from birth. A difference-in-differences analysis compared changes in VLBW infant outcomes between adopting and nonadopting NICUs before and after hospital-level adoption of probiotics. Additional analyses used the proportion of neonates treated with probiotics in each neonate’s birth NICU and year.

**Results:**

The analysis included 307 905 neonates with VLBW (mean [SD] gestational age, 28.4 [2.9] weeks; 50.0% male) at 807 US hospitals. The rate of probiotic treatment of neonates with VLBW rose from 1572 of 38 296 neonates (4.1%) in 2012 to 4788 of 37 910 (12.6%) in 2019. Only 123 of 745 NICUs (16.5%) adopted probiotics by 2019, with 4591 of 6017 neonates with VLBW (76.3%) receiving probiotics in 2019 at adopting NICUs. Incidence of NEC declined by 18% at adopting NICUs (odds ratio [OR], 0.82; 95% CI, 0.70-0.95; *P* = .10) compared with nonadopting NICUs. Probiotic adoption was not associated with a significant reduction in sepsis (OR, 1.11; 95% CI, 0.98-1.25; *P* = .09) or mortality (OR, 0.93; 95% CI, 0.80-1.08; *P* = .33).

**Conclusion and Relevance:**

In this cohort study, adoption of routine use of probiotics increased slowly in US NICUs and was associated with lower NEC risk but not with sepsis or mortality among neonates with VLBW. The findings for probiotic adoption and NEC, sepsis, and mortality were smaller than would have been predicted by the totality of RCT evidence but are consistent with a meta-analysis restricted to studies at low risk of bias.

## Key Points

**Question:**

Did probiotic use change in US neonatal intensive care units (NICUs) between 2012 and 2019, and is use of probiotics associated with improved health outcomes in neonates with very low birth weight (VLBW)?

**Findings:**

In this cohort study of 307 905 neonates with VLBW in 807 NICUs from 2012 to 2019, 17% of NICUs had adopted routine use of probiotics by 2019. Incidence of necrotizing enterocolitis declined by 18% at adopting NICUs vs nonadopting NICUs, and probiotic adoption was not associated with significant changes in mortality or sepsis.

**Meaning:**

In this study, probiotic use increased in US NICUs, and probiotic use was associated with a decline in necrotizing enterocolitis but not with sepsis or mortality rates.

## Teaser

This cohort study examines changes in probiotic use among neonates with very low birth weight in US neonatal intensive care units (NICUs) between 2012 and 2019 and the association of routine probiotic use in this population with health outcomes.

## Introduction

Although the incidence of necrotizing enterocolitis (NEC) has decreased, affected preterm infants remain at high risk for death, neurodevelopmental disabilities, repeated surgeries, and long-term tube feeding.^1,2^ Three recent meta-analyses, summarizing 30 to 56 randomized clinical trials (RCTs), evaluated use of enteral probiotics among infants with very low birth weight (VLBW).^3,4,5^ These studies found that probiotics were associated with a 43% to 45% reduction in NEC, 11% to 14% reduction in sepsis, and 23% to 24% reduction in mortality,^3,4,5^ although sensitivity analyses of trials with low risk of bias found more modest changes in NEC and no significant associations with sepsis or mortality.^5^ In response to this research, there have been recent calls for widespread adoption of probiotics in newborn intensive care units (NICUs).^6,7,8,9^ Nevertheless, the American Academy of Pediatrics does not recommend universal administration of probiotics to preterm infants given the lack of US Food and Drug Administration (FDA)–regulated products and conflicting safety and efficacy data.^10^

There is little evidence on how US NICUs have responded to this research and the conflicting expert guidance. Both the extent of probiotic adoption and the effectiveness of probiotic treatment under clinical conditions remain unknown. There is additional clinical uncertainty about the benefits of probiotic supplementation for infants with extremely low birth weight (ELBW),^5^ and the benefits may depend on whether infants are exposed to beneficial bacteria or probiotics through vaginal delivery^11^ or breast milk^12^ in the absence of supplementation. A 2015 survey suggested that there were low levels of probiotic adoption in NICUs,^13^ but recent, large-scale estimates of probiotic use are not available.

As new treatments diffuse into practice, the benefits demonstrated in clinical trials may not always materialize. Effective treatments may diffuse slowly,^14^ and there may be a gap between efficacy under ideal conditions and effectiveness in practice.^15^ Effectiveness is a particular concern for probiotics, which have not been approved as a treatment by the FDA and for which production is less carefully regulated than would be the case for an approved drug.^7^ Monitoring the health effects of probiotics as they diffuse into practice is a crucial component of evaluating their safety and effectiveness.

This study investigated the diffusion and clinical benefits of probiotic use among neonates with VLBW in NICUs when probiotic adoption occurred outside a clinical trial context. We used data from NICUs in the US to study changes in the rates of NEC, sepsis, and mortality, comparing the experiences of NICUs that did and did not adopt probiotics.

## Methods

### Data

This cohort study used data from the Vermont Oxford Network (VON), a voluntary learning community dedicated to improving newborn care. Research in support of the VON mission is funded by membership fees. VON has members in 49 states and is estimated to include data on over 85% of infants with VLBW born in the US.^15^ The University of Vermont’s institutional review board determined that use of VON’s deidentified research repository for this analysis was not human participants research and thus deemed the study exempt from institutional review board approval, with a waiver of informed consent. This study followed the Strengthening the Reporting of Observational Studies in Epidemiology (STROBE) reporting guideline for cohort studies.

The analysis included all eligible VON members in the US. Participating centers are listed in eTable 5 in Supplement 1. Member NICUs submit standardized data to VON on all neonates with VLBW treated at their facility within 28 days of birth. We used data for all neonates born weighing 501 g to 1500 g from January 1, 2012, to December 31, 2019. The sample was limited to neonates with a length of hospital stay of at least 3 days and with nonmissing data on neonate characteristics, probiotic treatment status, and outcomes.

Probiotic use was defined as formulas containing probiotics or probiotic supplements added to formula or breast milk feeds. For each neonate, the data also included health outcomes (NEC, in-hospital mortality, and bacterial or fungal infection after day 3 from birth) and factors associated with those outcomes recorded at birth (sex, race and ethnicity, birth weight, gestational age, 1-minute Apgar score, multiple birth, congenital malformation, whether the neonate was small for gestational age, and whether the neonate was transferred to the NICU from an outside hospital).^16^ Detailed definitions of the variables can be found in the eMethods in Supplement 1.

Probiotic use at a neonate’s NICU was defined as the proportion of all other neonates born in the same year and admitted to the same NICU who were treated with probiotics. To study patterns of probiotic adoption across hospitals, we followed a long history of research on technology diffusion^17^ and defined initial adoption using a threshold level of probiotic use. Specifically, the year that a NICU first adopted probiotics was defined as the first year in our data when at least 20% of neonates with VLBW received probiotics.

### Statistical Analysis

The decision to treat an individual neonate with probiotics may be endogenously related to the neonate’s health status, length of NICU stay, and survival. To avoid confounded comparisons of neonates who received probiotics and those who did not, our cohort study used a difference-in-differences approach^18^ that compared neonate outcomes across adopting and nonadopting hospitals before and after hospital-level adoption of probiotics. This approach yielded unbiased estimates of probiotic treatment outcomes under the assumption that in the absence of probiotic adoption, adopting and nonadopting hospitals would have had parallel trends in the evolution of NEC, sepsis, and mortality outcomes.

Probiotic diffusion was characterized by the percentage of all neonates with VLBW receiving probiotics and the number of hospitals newly adopting probiotics over time. For hospitals that had adopted probiotics by 2019, we calculated the percentage of neonates receiving probiotics in those hospitals in the 4 years before and 4 years after adoption.

We used fixed-effects logistic regressions to estimate the association between neonate outcomes and hospital-level probiotic adoption. The unit of observation was at the neonate level. All regressions included hospital-level intercepts (fixed effects) to account for fixed differences in outcomes across hospitals and individual year effects to account for temporal trends in outcomes. All regressions further controlled for neonate factors associated with NEC, in-hospital mortality, and sepsis that have been previously validated, including birth weight, gestational age, small for gestational age, race and ethnicity, sex, multiple birth, location of birth, 1-minute Apgar score, and major birth defect.^19^ Race and ethnicity were reported by the mother if possible and otherwise from the birth certificate or medical record and categorized as American Indian or Alaska Native, Asian, Black, Hispanic, non-Hispanic White, and other (if none of the race and ethnicity categories applied).

To estimate changes in neonate outcomes each year before and after a hospital adopted probiotics, we conducted an event study analysis. Specifically, we estimated a fixed-effects logistic regression including indicator variables for each event year relative to the hospital’s adoption year; year 0 was defined as the first year that the NICU achieved at least 20% probiotic use. The coefficients on the event-year indicators estimated how neonate outcomes changed before and after probiotic adoption at adopting NICUs compared with changes at nonadopting units.

For our final analysis, we replaced the event-year variables with a single treatment variable that captured continuous NICU-level variation in the extent of probiotic adoption: the proportion of all other neonates with VLBW born in the same year and at the same NICU who were treated with probiotics. This analysis extended the logic of our difference-in-differences approach but used the continuous variation in NICU-level probiotic use, rather than dichotomizing adoption status. Regression coefficients directly scaled to estimate probiotic effects per treated neonate, which allowed us to compare the magnitude of our estimates directly with the RCT findings.^3,4,5^

In exploratory analyses, we interacted NICU-level probiotic use with 3 neonate characteristics to test whether probiotic use had different associations with health outcomes in subgroups: whether the neonate was born via cesarean delivery, received breast milk prior to discharge, or had ELBW (defined as <1000 g).

Statistical significance was set at *P* < .05 using a 2-sided *t* test of whether the odds ratio (OR) associated with NICU probiotic use was equal to 1, indicating no association between NICU probiotic use and neonate outcomes. Additional details about the estimation approach are given in the eMethods in Supplement 1. Data analysis was performed from January 2022 through February 2023, using Stata, version 15 (StataCorp LLC).

## Results

The sample included 307 905 neonates with VLBW with a length of stay of at least 3 days and nonmissing data on neonate characteristics, probiotic treatment status, and outcomes (mean [SD] gestational age, 28.4 [2.9] weeks; 50.0% female and 50.0% male; 0.8% American Indian or Alaska Native, 4.9% Asian, 30.3% Black, 18.2% Hispanic, 43.2% non-Hispanic White, and 2.0% other). These neonates were treated at 807 NICUs located in the US.

**Figure 1.**
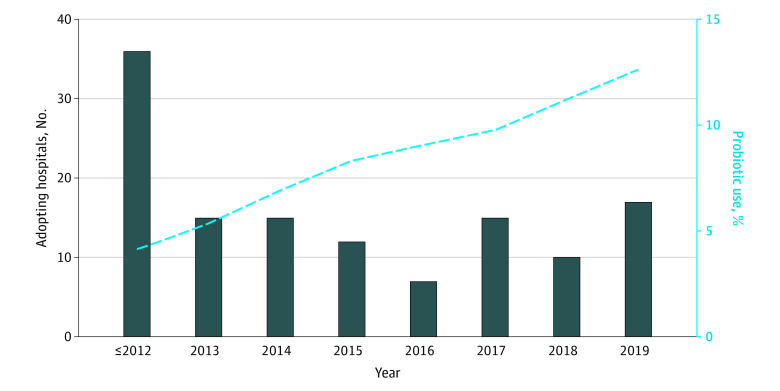
Trends in Probiotic Adoption From 2012 to 2019 Bars show the number of hospitals adopting probiotics in each year of the sample. The adoption year was defined as the earliest year that at least 20% of in-sample neonates with very low birth weight (VLBW) received probiotics. The dashed line shows the proportion of neonates with VLBW in the sample who received probiotics each year; this proportion was calculated using data from all in-sample hospitals regardless of probiotic adoption status.

Probiotic use diffused slowly over our study period, with use remaining low at most hospitals. Figure 1 shows that national probiotic use for neonates with VLBW rose from 1572 of 38 296 neonates (4.1%) in 2012 to 4788 of 37 910 (12.6%) in 2019. In 2012, only 36 of 645 hospitals (5.6%) treated at least 20% of their neonates with VLBW with probiotics; by 2019, 123 of 745 hospitals (16.5%) had achieved this threshold. At probiotic-adopting hospitals, 4591 of 6017 neonates with VLBW (76.3%) received probiotics in 2019.

The Table reports summary statistics contrasting neonates at 680 nonadopting NICUs, 91 newly adopting NICUs that exceeded 20% probiotic use for neonates with VLBW for the first time between 2013 and 2019, and 36 early-adopting NICUs that were already providing probiotic treatment to more than 20% of neonates with VLBW in 2012. Nonadopting NICUs reported a mean (SD) of 7.8 (0.9) years of neonate data over our 8-year sample period. For newly adopting NICUs, our data covered a mean (SD) of 3.6 (2.1) preperiod years and 4.1 (2.0) postperiod years. Infants at adopting and nonadopting NICUs had similar mean birth weight, gestational age, and 1-minute Apgar scores. Probiotic adoption rates were higher in the West census region and lower in the Northeast (eTable 1 in Supplement 1). Trends over time in probiotic use, NEC, sepsis, and mortality rates are reported in the eFigure in Supplement 1.

**Table.** Descriptive Statistics by NICU Probiotic Adoption Status

Characteristic	Neonates^a^		
	No probiotic adoption by 2019	Adopted probiotics in 2013-2019	Adopted probiotics in 2012 or earlier
Sample size			
NICUs, No.	680	91	36
Neonates, No.	258 194	33 580	16 131
Neonatal care			
Received probiotics	863 (0.3)	12 696 (37.8)	12 336 (76.5)
Cesarean delivery	192 080 (74.4)	25 440 (75.8)	12 149 (75.3)
Any breast milk	124 940 (48.4)	19 372 (57.7)	7357 (45.6)
Neonatal outcomes			
Necrotizing enterocolitis	12 737 (4.9)	1584 (4.7)	567 (3.5)
Sepsis	24 752 (9.6)	2905 (8.7)	1589 (9.9)
Mortality	14 698 (5.7)	1998 (6.0)	880 (5.5)
Neonatal characteristics			
Multiple birth	67 812 (26.2)	9046 (26.9)	4144 (25.7)
Major birth defect	11 113 (4.3)	1614 (4.8)	773 (4.8)
Extremely low birth weight^b^	100 326 (38.9)	12 933 (38.5)	6360 (39.4)
Birth weight, mean (SD), g	1078 (279.2)	1081 (277.6)	1073 (279.4)
Gestational age, mean (SD), wk	28.4 (2.9)	28.4 (2.9)	28.3 (2.8)
1-min Apgar score, mean (SD)	5.3 (2.5)	5.3 (2.4)	5.3 (2.4)
Sex			
Female	128 775 (49.9)	17 081 (50.9)	8168 (50.6)
Male	129 419 (50.1)	16 499 (49.1)	7963 (49.4)
Race and ethnicity			
American Indian or Alaska Native	1875 (0.7)	467 (1.4)	58 (0.4)
Asian	11 752 (4.6)	2625 (7.8)	837 (5.2)
Black	82 855 (32.1)	6215 (18.5)	4119 (25.5)
Hispanic	44 653 (17.3)	8077 (24.0)	3328 (20.6)
Non-Hispanic White	109 854 (42.6)	15 606 (46.5)	7433 (46.1)
Other^c^	5494 (2.1)	431 (1.3)	266 (1.7)
Sample coverage over time, per NICU			
Total years in sample, mean (SD), No.	7.8 (0.9)	7.7 (0.8)	8.0 (0.3)
Preadoption years in sample, mean (SD), No.	NA	3.6 (2.1)	0
Postadoption years in sample, mean (SD), No.	NA	4.1 (2.0)	8.0 (0.3)

Abbreviations: NA, not applicable; NICU, neonatal intensive care unit.

^a^ Data are presented as the number (percentage) of neonates unless otherwise indicated.

^b^ Defined as less than 1000 g.

^c^ Respondents were instructed to report race and ethnicity as “other” if none of the other categories applied.

**Figure 2.**
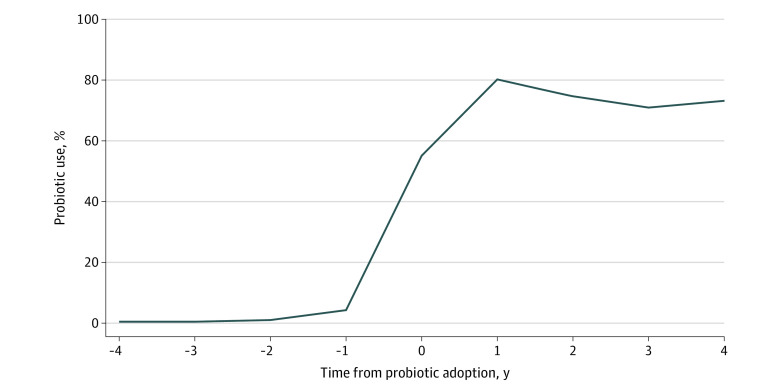
Rates of Probiotic Use Among Neonates With Very Low Birth Weight (VLBW) Before and After the Probiotic Adoption Year Among Adopting Hospitals Year 0 is defined as the probiotic adoption year, the earliest year when at least 20% of in-sample neonates with VLBW received probiotics at a hospital. The sample included hospitals that first adopted probiotics between 2013 and 2019.

Figure 2 plots probiotic use at adopting hospitals relative to the year of probiotic adoption (the first year with ≥20% use within the NICU); this year is indicated as year 0 in the graph. Probiotic use remained below 5% in the years prior to adoption and climbed steeply thereafter, reaching 80% in year 1.

**Figure 3.**
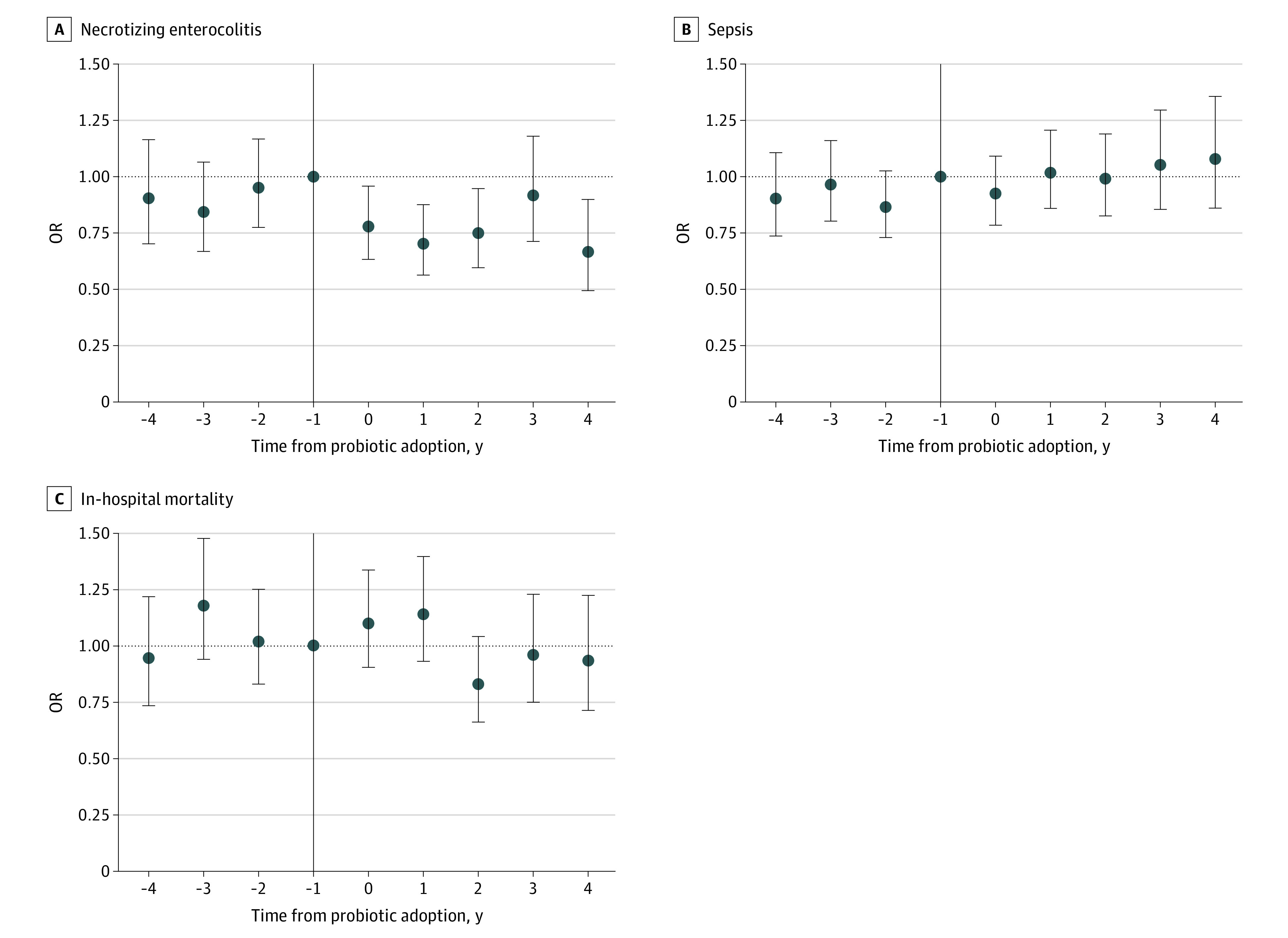
Event Study Analysis of Neonatal Outcomes Before and After Hospital Probiotic Adoption Compared With Trends at Nonadopting Hospitals Among 307 905 Neonates The plotted points are regression coefficients on a series of indicator variables for years before and after the hospital first reached at least 20% probiotic use (normalized to year 0); whiskers indicate 95% CIs. All regressions control for calendar-year fixed effects, hospital fixed effects, and neonate characteristics (birth weight, gestational age, small for gestational age, race and ethnicity, sex, multiple birth, location of birth, 1-minute Apgar score, and major birth defect). The odds ratio (OR) for 1 to 4 years after adoption vs 1 to 4 before adoption was 0.82 (95% CI, 0.70-0.95) for necrotizing enterocolitis (A), 1.11 (95% CI, 0.98-1.25) for sepsis (B), and 0.93 (95% CI, 0.80-1.08) for in-hospital mortality. Additional details are given in the eMethods in Supplement 1.

Figure 3 plots difference-in-differences event-study estimates of the change in neonate outcomes in each year before and after hospitals adopted probiotics compared with hospitals that did not adopt probiotics. The indicator variable for the year immediately preceding adoption (event year −1) was omitted from the regression and used as the reference category. The incidence of NEC declined by 18% at adopting NICUs (OR, 0.82; 95% CI, 0.70-0.95; *P* = .01) compared with trends at nonadopting hospitals, differencing the mean regression coefficient for the postevent years (0 through 3) from the mean coefficient for the pre-event years (–4 through –1). Figure 3B and C show that probiotic adoption was not associated with any significant reduction in sepsis (OR, 1.11; 95% CI, 0.98-1.25; *P* = .09) or mortality (OR, 0.93; 95% CI, 0.80-1.08; *P* = .33).

Figure 3 also allowed us to assess whether adopting and nonadopting hospitals were on parallel trends prior to probiotic adoption. The stable preperiod coefficients suggest that there were not differential trends at adopting hospitals prior to probiotic adoption. For each health outcome, an *F* test of the joint significance of the preperiod relative-year coefficients found that they were not statistically distinguishable from 0 (Figure 3).

**Figure 4.**
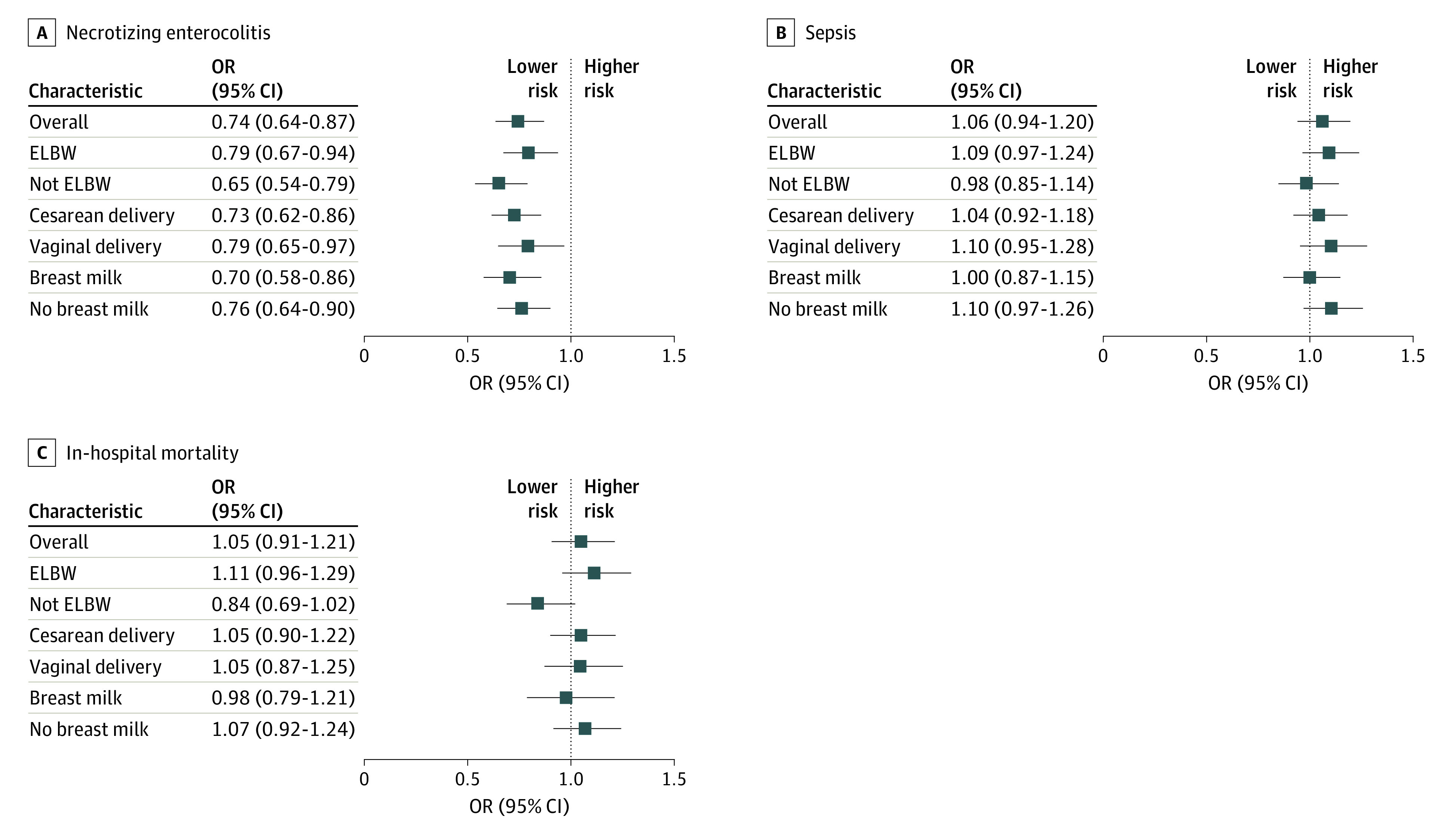
Association Between Hospital-Level Probiotic Use and Neonate Outcomes Among 307 905 Neonates Estimated effects from logit regressions are shown. The independent variable of interest in the overall regressions was the rate of probiotic use among other neonates in the same neonatal intensive care unit–year. For the subsequent specifications, the probiotic use rate was interacted with neonate characteristics. All regressions controlled for calendar-year fixed effects, hospital fixed effects, and neonate characteristics (birth weight, gestational age, small for gestational age, race and ethnicity, sex, multiple birth, location of birth, 1-minute Apgar score, and major birth defect). Data markers represent point estimates, and error bars indicate 95% CIs. Additional details are given in the eMethods in Supplement 1. ELBW indicates extremely low birth weight and OR, odds ratio.

Figure 4 plots estimates from models using the proportion of neonates receiving probiotics in each hospital and year as the exposure variable; detailed results are reported in eTable 2 in Supplement 1. Probiotic treatment was associated with lower rates of NEC, with an OR of 0.74 (95% CI, 0.64-0.87; *P* < .001). There was no significant association of probiotic exposure with sepsis risk (OR, 1.06; 95% CI, 0.94-1.20; *P* = .34) or mortality (OR, 1.05; 95% CI, 0.91-1.21; *P* = .52). To compare these results to meta-analyses reporting relative risk ratios (RRs), we rescaled the ORs using the mean outcome risk in nonadopting hospitals: NEC with an RR of 0.75 (95% CI, 0.65-0.88); sepsis with an RR of 1.05 (95% CI, 0.94-1.18); and mortality with an RR of 1.05 (95% CI, 0.91-1.21).

Our approach relied on changes over time in NICU-level probiotic adoption rates rather than a conventional analysis directly comparing treated and untreated neonates. Direct comparisons of treated and untreated neonates would likely have been biased by unobserved confounding variables. Infants treated with probiotics had lower birth weight and Apgar scores and younger gestational age and were more likely to have a major birth defect (eTable 3 in Supplement 1). In contrast, changes in hospital-level probiotic use were not associated with changes in these factors (eTable 3 in Supplement 1), suggesting that our difference-in-differences analysis was less subject to confounding.

Figure 4 reports results of exploratory tests for heterogeneity in the association of unit-level probiotic adoption with neonate outcomes depending on whether the neonate had ELBW (birth weight, <1000 g), was born via cesarean delivery, or received any breast milk at discharge. Probiotics were estimated to confer smaller benefits for neonates with ELBW compared with those who did not have ELBW. The association of probiotics with neonate outcomes was not significantly different for neonates delivered vaginally or via cesarean or for neonates who did or did not receive breast milk.

In sensitivity analyses, we restricted to NICUs that remained in the sample for every year of the study period (eTable 4 in Supplement 1) and excluded NICUs that had already achieved 20% adoption of probiotics by 2012 (eTable 4 in Supplement 1) and found similar results. We also confirmed similar results for our binary adoption analysis when using a 10% (rather than 20%) probiotic use threshold to define adoption (eTable 4 in Supplement 1).

## Discussion

In this cohort study of 807 NICUs, only 12.6% of neonates with VLBW were receiving probiotics and only 16.5% of NICUs had adopted probiotic treatment as of 2019. Initially, these low rates of probiotic diffusion present a puzzle. A recent Cochrane review and meta-analysis of more than 50 published RCTs studying the outcomes of probiotics for infants with VLBW found that this body of evidence suggests benefits of probiotics for NEC, sepsis, and mortality, although the evidence was rated as low certainty due to small trial sample sizes and unreliable methods used in many of the trials.^5^

Our findings suggest that NICU adoption of probiotics was associated with smaller benefits than those found in meta-analyses of RCTs.^3,4,5^ The NEC reduction associated with infant-level treatment with probiotics in clinical trials (RR, 0.54 [95% CI, 0.45-0.65])^5^ was larger than our estimated reduction (RR, 0.75 [95% CI, 0.65-0.88], based on our results in Figure 4 and rescaling OR to RR) and even lay outside the 95% CI for our estimate. Similarly, in the trial meta-analysis, the RRs of probiotics for sepsis (0.89 [95% CI, 0.82-0.97]) and mortality (0.76 [95% CI, 0.65-0.89])^5^ were larger than our estimates for these outcomes (sepsis: RR, 1.05; 95% CI, 0.94-1.18; mortality: RR, 1.05; 95% CI, 0.91-1.21) and lay outside our 95% CIs. Both the meta-analysis and our own analysis found weak evidence that probiotics benefit neonates with ELBW compared with neonates with VLBW.

There are many reasons why probiotics may be less effective in practice than in published clinical trials. First, prior research suggests risk of bias in the published studies on probiotics.^5,20^ In the Cochrane review,^5^ sensitivity meta-analyses of trials at low risk of bias did not show associations with mortality or infection and found smaller reductions in NEC risk (RR, 0.70 [95% CI, 0.55-0.89]), more in line with our findings. Second, patients enrolled in trials may differ systematically from patients treated in a NICU, although we found limited evidence of heterogeneity in the association between outcomes and probiotic adoption across neonate subgroups. Finally, other practices that are associated with the effectiveness of probiotics may differ from the RCT context. Probiotics used in trials and in practice may vary in timing, dose, and formulation of the probiotic feedings. This may be particularly true in US NICUs, where probiotics have not been approved by the FDA and production is less carefully regulated than for an approved drug.^10^

Studying the impact of probiotics (and other medical innovations^21^) as they diffuse into clinical practice through postmarket surveillance is critical to developing a body of evidence on benefits in general clinical practice. Our analysis demonstrates that the difference-in-differences approach can provide a useful framework for estimating treatment outcomes in observational data in settings where patient-level comparisons may be confounded.

Uncertainty about the quality of commercially available probiotics may contribute to lagging adoption rates. Developing direct evidence of their outcomes in practice, as we did in this study, is an important step toward establishing safety and efficacy for routine use. Another possible avenue to increased adoption may be the commercial entry of an FDA-regulated probiotic product. We speculate that a major hurdle is that probiotic formulations cannot easily gain patent protection^22^; by protecting the entrant’s initial monopoly power, patent protection can be a crucial incentive to encourage costly market entry.^23^ Without the safety, quality, and efficacy assurances that accompany the FDA drug review process, NICUs are left to interpret evidence and assure product integrity independently. Our results suggest lessening barriers to probiotic adoption and widening access could spur important reductions in NEC for neonates with VLBW.

### Limitations

This study has limitations. As with all observational studies, the association between probiotic adoption and outcomes for neonates with VLBW could be confounded by unmeasured factors associated with hospital adoption of probiotics. The difference-in-differences approach does not rely on individual-level comparisons of treated and untreated neonates within the same NICU and year, where treatment choices are likely confounded by differences in neonate health status. The model also accounted for differences across hospitals that were fixed over time but did not account for time-varying factors that changed at the hospital level. Because our event-study analysis suggests that there were no differential trends in patient outcomes prior to adoption of probiotics, and changes in patient outcomes for NEC occurred at the same time as adoption of probiotics, any time-varying confounder would need to have coincided with probiotic adoption. For example, a harmful treatment could confound our estimates if it was always adopted at the same time as probiotics and tended to offset the benefits, although there is no direct evidence of such a confounder.

We were not able to investigate many potential reasons for the gap between the efficacy of probiotics in clinical trials and the effectiveness of probiotics as they have diffused in practice. The VON data do not track the timing, dose, or formulation of the probiotic feedings, each of which may influence the potential benefits of this treatment.^24,25^ We did not know the timing of NEC or sepsis relative to probiotic administration; both were defined as occurring at any time and not necessarily at the specific hospital where probiotics were given. Efficacy may also depend on each neonate’s preexisting gut microbiome. Investigating these factors remains a crucial avenue for future research.

## Conclusions

In this cohort study of outcomes for neonates with VLBW in NICUs that adopted routine probiotic supplementation and those that did not adopt probiotics, we found that probiotic adoption was associated with lower NEC risk but not lower risk of sepsis or mortality among neonates with VLBW. The associations of probiotic adoption with NEC, sepsis, and mortality were smaller than would have been predicted based on clinical trial evidence,^5^ although they were consistent with a meta-analysis restricted to trials at low risk of bias.^5^ These findings highlight the importance of monitoring the effectiveness of probiotics as they diffuse into neonatal practice beyond the setting of clinical trials. The analysis also suggests a framework that might be applicable to postapproval effectiveness evaluations for other new clinical treatments, providing insight into how a technology affects patient outcomes as it diffuses into routine clinical practice.

## Notes

Supplement 1.
eMethods
**eFigure.** Trends Over Time in Probiotics Use, NEC, Sepsis, and Mortality by NICU Adoption Status **eTable 1.** Descriptive Data on Characteristics of Nonadopting, Newly Adopting, and Early-Adopting Hospitals **eTable 2.** Complete Regression Results and Standard Errors for Specifications Graphed in Figure 4 **eTable 3.** Association of Infant Risk Factors With Infant and NICU Use of Probiotics **eTable 4.** Alternative Regression Specifications, Varying Sample and Definition of Probiotic Adoption **eTable 5.** Vermont Oxford Network Members

Supplement 2.
Data Sharing Statement
