# Supplementary material for: Association of Hospital Adoption of Probiotics With Outcomes Among Neonates With Very Low Birth Weight
Source: JAMA Health Forum. 2023 May 12;4(5):e230960. doi: 10.1001/jamahealthforum.2023.0960 (PMC10182437; doi:10.1001/jamahealthforum.2023.0960)
Supplement: Supplement 1. — eMethods eFigure. Trends Over Time in Probiotics Use, NEC, Sepsis, and Mortality by NICU Adoption Status eTable 1. Descriptive Data on Characteristics of Nonadopting, Newly Adopting, and Early-Adopting Hospitals eTable 2. Complete Regression Results and Standard Errors for Specifications Graphed in Figure 4 eTable 3. Association of Infant Risk Factors With Infant and NICU Use of Probiotics eTable 4. Alternative Regression Specifications, Varying Sample and Definition of Probiotic Adoption eTable 5. Vermont Oxford Network Members [file jamahealthforum-e230960-s001.pdf]

Supplementary Online Content

Agha L, Staiger D, Brown C, Soll RF, Horbar JD, Edwards EM. Association of hospital adoption of probiotics with outcomes among neonates with very low birth weight. *JAMA Health Forum*. 2023;4(5):e230960. doi:10.1001/jamahealthforum.2023.0960

eMethods
eFigure. Trends Over Time in Probiotics Use, NEC, Sepsis, and Mortality by NICU Adoption Status
eTable 1. Descriptive Data on Characteristics of Nonadopting, Newly Adopting, and Early-Adopting Hospitals
eTable 2. Complete Regression Results and Standard Errors for Specifications Graphed in Figure 4
eTable 3. Association of Infant Risk Factors With Infant and NICU Use of Probiotics
eTable 4. Alternative Regression Specifications, Varying Sample and Definition of Probiotic Adoption
eTable 5. Vermont Oxford Network Members

This supplementary material has been provided by the authors to give readers additional information about their work.

© 2023 Agha L et al. *JAMA Health Forum*.

eMethods

Data Appendix: Definitions of Key Outcome Variables

Necrotizing enterocolitis is defined by the VON as diagnosed at surgery, at postmortem examination, or with clinical and radiographically using the criteria below.

1. At one of the following clinical signs present:
    a. bilious gastric aspirate or emesis
    b. abdominal distension or discoloration
    c. occult or gross blood in stool (no fissure);
2. AND at least one of the following radiographic findings present:
    a. pneumatosis intestinalis
    b. hepato-biliary gas
    c. pneumoperitoneum.
3. Infants who satisfy the definition of NEC above but are found to have a focal intestinal perforation at surgery or postmortem examination should be coded as having focal intestinal perforation, not NEC.

Note that the alternative diagnosis of Spontaneous Intestinal Perforation (SIP) is recorded separately. SIP is defined as a focal intestinal perforation separate from necrotizing enterocolitis. The diagnosis will be based on visual inspection of the bowel at the time of surgery or post-mortem examination that demonstrates a single focal perforation with the remainder of the bowel appearing normal. Infants with SIP were not included in this analysis. Despite standardized definitions, misclassification could occur, although it is unlikely that enough infants were misclassified to influence the results.

Sepsis is defined as late infections occurred after day 3 from birth and included: bacterial pathogens on a specified list recovered from blood or cerebrospinal fluid; coagulase negative Staphylococcus recovered from blood or cerebrospinal fluid plus one or more sign(s) of infection and treatment with at least five days of intravenous antibiotics; or fungus recovered from a blood culture. Infants could have more than one type of infection.

Mortality is defined as all-cause, in-hospital mortality. Note that because our sample excludes infants with length of stay shorter than 3 days, infants that die within 2 days of birth are excluded from this analysis. Mortality immediately following birth is unlikely to be affected by probiotics. This definition is similar to the definitions used in many of the clinical trials, which followed all-cause mortality before discharge from hospital as a primary outcome.[1] In clinical trials, probiotics were typically initiated in the first week of life, often with the first enteral feed.

© 2023 Agha L et al. *JAMA Health Forum*.

Statistical Methods Appendix

In this paper, we estimate several different statistical models, all adapted from a difference-in-differences approach. The difference-in-differences approach compares changes in infant outcomes at adopting NICUs to changes in infant health outcomes at non-adopting NICUs. The unit of observation for all models is an individual infant.

The first set of estimates uses an event-study framework to compare trends in infant health outcomes across adopting and non-adopting hospitals. We denote health outcomes Y_{iht} (including mortality, necrotizing enterocolitis, and sepsis) for infant i admitted to hospital NICU h at time t. Further, define $r(h,t)$ as the event year for an adopting hospital, with $r(h,t) = -1$ in the year before adoption, $r(h,t) = 0$ during the first year of adoption, and so on. We estimate fixed effect logit regressions of the following form:

$$\Pr(Y_{iht} = 1) = F(\theta_{r(h,t)} Adopt_h + \delta_h + \gamma_t + \lambda X_i) \qquad (1)$$

The variable $Adopt_h$ equals 1 for hospitals that adopt probiotics between 2013-2019, and equals 0 for all other hospitals. The vector $\theta_{r(h,t)}$ identifies the comparison of adopting to non-adopting hospitals in relative event year $r(h,t)$. We exclude the $r(h,t) = -1$ category from the regression estimation, so that the comparison θ_{-1} is normalized to zero in the year prior to adoption. Non-adopting hospitals and hospitals that adopted in 2012 or earlier have $\theta_{r(h,t)} = 0$. The regression includes hospital fixed effects δ_h to allow for fixed differences across hospitals in patient outcomes. The regression also includes year fixed effects γ_t. The inclusion of hospital and year fixed effects ensures that variation identifying adoption effects comes from within-hospital changes in infant outcomes, comparing trends in adopting vs. non-adopting hospitals. Finally the regression includes infant characteristics X_i including birth weight, gestational age, SGA, race, sex, multiple, location of birth, 1-minute APGAR score, major birth defect. These controls help account for potential time-varying differences in patterns of patient sorting across NICUs.

We plot the coefficients $\theta_{r(h,t)}$ in Figure 3. These plots allow us to assess the parallel trends assumption visually. If health outcomes at adopting and non-adopting hospitals have parallel trends, then we would expect no differential changes in infant outcomes over the pre-period at adopting hospitals. We formalize this with an F-test for whether the values $\theta_{r(h,t)}$ are jointly equal to 0 over the pre-period, i.e. for all $r < -1$. We also use the specification in equation (1) to calculate a single estimate of association between NICU-level probiotic use and infant outcomes: we difference the average value of $\theta_{r(h,t)}$ for $1 \leq r \leq 4$ with the average value of $\theta_{r(h,t)}$ for $-4 \leq r \leq -1$. This estimates the average effect of probiotic adoption, comparing the four years after adoption to the four years before.

Our second specification makes use of the continuous variation in probiotic use across hospitals and over time, thus accounting for the fact that we would expect a smaller decline in NEC at a hospital where only 20% of VLBW infants receive probiotics relative and a larger decline at a hospital where 80% of VLBW infants receive probiotics. We estimate a fixed effect logit regression of the following form:

$$\Pr(Y_{iht} = 1) = F(\beta PctProbiotics_{-i,ht} + \delta_h + \gamma_t + \lambda X_i) \qquad (2)$$

The treatment variable $PctProbiotics_{-i,ht}$ calculates the proportion of infants treated in that hospital-year who receive probiotics, excluding the index infant; this proportion ranges can range from 0 (no infants receive probiotics) to 1 (all infants receive probiotics). We exclude the index infant from this calculation to avoid bias that can arise from correlation between the infant's own health status and the decision to treat that infant with probiotics.[1] As before, the regression includes hospital fixed effects δ_h, year fixed effects γ_t, and a vector of infant characteristics X_i. β estimates the effect of probiotics on treated infants, and is identified from within-hospital over-time variation in the hospital-level probiotic

[1] This bias is small at hospitals that treat a large number of infants each year, so that the influence of any single infant on the hospital-level $PctProbiotics_{ht}$ is negligible. Consistent with this observation, our results are not sensitive to whether or not we exclude the index infant from the calculation of hospital-level probiotic use.

© 2023 Agha L et al. *JAMA Health Forum*.

utilization rate, relative to over-time changes at non-adopting hospitals. Results from this specification are plotted in Figure 4, in the top row "Overall" results.

To investigate potential heterogeneity in treatment effects, we consider three further modifications to this specification that interact the $PctProbiotics_{-i,ht}$ variable with an infant level characteristic. For example, we estimate:

$$\begin{aligned}\Pr(Y_{iht} = 1) = F(&\beta_1 ELBW_i PctProbiotics_{-i,ht} + \beta_2(1 - ELBW_i)PctProbiotics_{-i,ht} \\ &+\delta_h + \gamma_t + \lambda X_i) \qquad (3)\end{aligned}$$

The variable $ELBW_i$ equals 1 if the infant is extremely low birth weight and equals 0 otherwise. This specification allows us to test whether the benefits of being treated at a hospital with high probiotic use are different for ELBW infants. Note that $ELBW_i$ is included among the infant characteristics X_i, in all specificaitons. In two additional regressions, we similarly test whether infants born by Cesarean section vs. vaginal deliveries have different benefits of being treated at a hospital with high probiotic use, and whether infants receiving any breast milk vs. formula alone have different benefits of being treated at a hospital with high probiotic use. (Recall that mode of delivery and breastmilk exposure are also among the control variables included in X_i.) These results are reported in the bottom three panels of Figure 4 and in eTable2.

To investigate whether the composition of infants treated at adopting NICUs is changing around the time of probiotic adoption, we estimate linear regressions of the following form:

$$X_i = \beta PctProbiotics_{-i,ht} + \delta_h + \gamma_t + \varepsilon_{iht}$$

This equation tests whether infant characteristics are changing differentially at adopting NICUs relative to non-adopting NICUs, in a way that is correlated with the timing of probiotic adoption. Although we can directly control for any such changes in observable infant characteristics, we use this test as a proxy for investigating potential selection on unobservable characteristics. Results are reported in eTable 3 column 1.

We contrast our approach focusing on hospital-level probiotic adoption with an alternative approach that investigates patterns of probiotic use within adopting NICUs, using the following equation:

$$X_i = \beta Probiotics_i + \delta_h + \gamma_t + \varepsilon_{iht}$$

This equation tests whether infants receiving probiotics at adopting NICUs are similar in their observable characteristics to infants who do not receive probiotics. This specification highlights the potential for confounding factors to bias any direct comparisons of treated and untreated infants. These results are reported in eTable 3 column 2.

We test alternative specifications in eTable 4. In Panel A we report our original estimates of equation 2 for comparison. In Panel B, we re-estimate equation 2, with a new sample restriction to include only hospitals that provide registry data to the VON in all eight years. In Panel C, we re-estimate equation 2, now eliminating early adopting hospitals that already treat at least 20% of their infants with probiotics as of 2012, the earliest year in our data. In Panel D, we estimate a simple difference-in-differences regression with two-way fixed effects. This is a variation on estimating equation (2) above, but replacing the continuously varying $PctProbiotics_{-i,ht}$ variable with a binary variable $PostAdoption20_{ht}$ which equals 1 if the hospital has crossed the 20% probiotic adoption threshold in the current year or any prior year. $PostAdoption20_{ht}$ equals 0 prior to probiotic adoption, and is always 0 for non-adopting hospitals. Results with the binary adoption variable are similar to the specifications using continuous variation in adoption status, which is consistent with the high rates of probiotic use among adopting hospitals. We also investigate the robustness of these results to using a different threshold of probiotic use to define adoption. In Panel E, we repeat the analysis but define a hospital as post-adoption if it has provided probiotics to at least 10% of VLBW infants in the current year or any prior year.

Appendix References

1. Sharif S, Meader N, Oddie SJ, Rojas-Reyes MX, McGuire W. Probiotics to prevent necrotising enterocolitis in very preterm or very low birth weight infants. *Cochrane Database of Systematic Reviews*. 2020;(10).

© 2023 Agha L et al. *JAMA Health Forum*.

eFigures and eTables

eFigure. Trends over time in probiotics use, NEC, sepsis and mortality by NICU adoption status

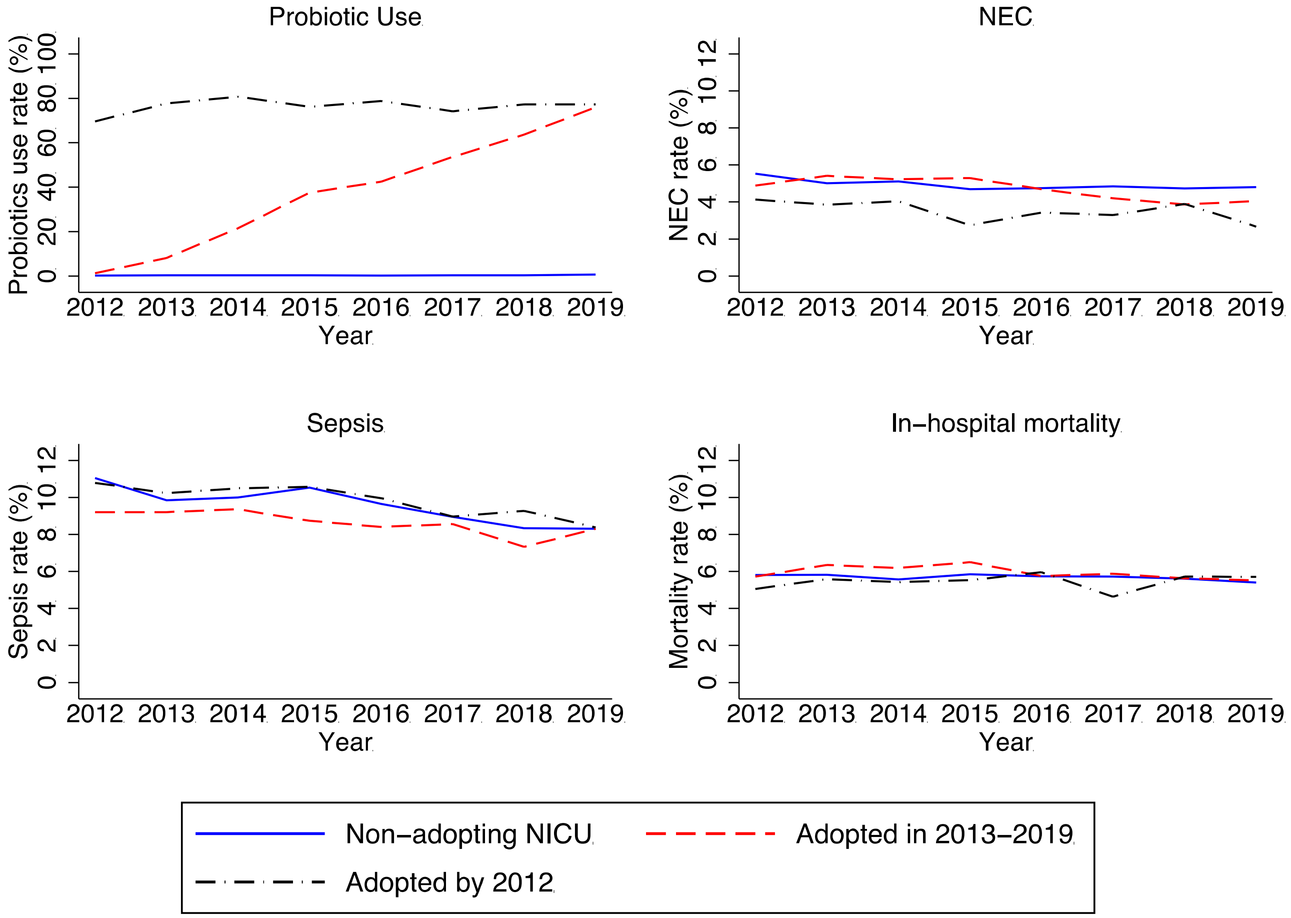

© 2023 Agha L et al. *JAMA Health Forum*.

eTable 1. Descriptive data on characteristics of non-adopting, newly-adopting, and early adopting hospitals

	No probiotic adoption by 2019	Adopted probiotics 2013 - 2019	Adopted probiotics 2012 or earlier
Mean number of admissions (per unit-year)	489	475	520
Pediatric residency	212 (34.1)	16 (19.3)	13 (39.4)
Region			
Midwest	111 (17.9)	9 (10.8)	9 (27.3)
Northeast	234 (37.6)	6 (7.2)	1 (3.0)
South	136 (21.9)	19 (22.9)	14 (42.4)
West	141 (22.7)	49 (59.0)	9 (27.3)
No. of NICUs reporting hospital characteristics	622	83	33

Note: Since not all NICUs submitting infant-level data also submit a complete hospital survey, the data summarized above covers 738 out of the 807 hospitals included in our main analysis.

© 2023 Agha L et al. *JAMA Health Forum*.

eTable 2. Complete regression results and standard errors for specifications graphed in Figure 4

	Dependent variable:		
	**Necrotizing enterocolitis**	**Sepsis**	**In-hospital mortality**
Independent variable:			
	A. Continuous adoption variable		
Proportion of VLBW infants given probiotics	0.743***	1.061	1.048
	(0.060)	(0.065)	(0.078)
	B. Continuous adoption interacted with birthweight		
ELBW x (Prop. of VLBW infants given probiotics)	0.794***	1.093	1.112
	(0.067)	(0.070)	(0.085)
Non-ELBW x (Prop. of VLBW infants given probiotics)	0.651***	0.984	0.839*
	(0.064)	(0.074)	(0.084)
	C. Continuous adoption interacted with Cesarean delivery		
Cesarean delivery x (Prop. of VLBW given probiotics)	0.727***	1.043	1.046
	(0.061)	(0.067)	(0.080)
Vaginal delivery x (Prop. of VLBW given probiotics)	0.792***	1.1041	1.045
	(0.081)	(0.082)	(0.096)
	D. Continuous adoption interacted with receiving any breastmilk		
Breastmilk x (Prop. of VLBW given probiotics)	0.704***	1.000	0.977
	(0.071)	(0.070)	(0.107)
No Breastmilk x (Prop. of VLBW given probiotics)	0.762***	1.104	1.067
	(0.065)	(0.073)	(0.083)

Notes: This table reports results (expressed as odds ratios) and standard errors from 12 separate logit regressions; these are the same results displayed graphically in Figure 4. The outcome variable of the logit regression listed in the column headers (NEC, sepsis, or mortality). The independent variable of interest in Panel A is the rate of probiotic use among other VLBW infants in the same NICU-year. For specifications reported in Panels B,C, and D, the probiotic use rate is interacted with infant characteristics. All regressions control for calendar year fixed effects, hospital fixed effects, and neonate characteristics (birth weight, gestational age, SGA, race, sex, multiple, location of birth, 1-minute APGAR score, and major birth defect). Sample size: 307,905 infants. ***indicates statistical significance at the 1% level. *indicates statistical significance at the 10% level.

© 2023 Agha L et al. *JAMA Health Forum*.

eTable 3. Association of infant risk factors with infant and NICU use of probiotics

	Independent variable:	
	Proportion VLBW infants in NICU given probiotics	Infant was given probiotics
	(1)	(2)
Dependent variable:		
Multiple	-0.001	0.005
	(0.010)	(0.006)
Birthweight	-3.661	-65.822***
	(6.530)	(11.088)
Extremely low birth weight (<1000g)	0.002	0.099***
	(0.011)	(0.019)
Gestational age	-0.013	-0.905***
	(0.061)	(0.121)
APGAR score (1 min)	0.087	-0.194***
	(0.061)	(0.059)
Major birth defect	-0.002	-0.012***
	(0.004)	(0.003)
Sex - male	0.008	0.014***
	(0.008)	(0.005)
Race - black	0.002	0.002
	(0.008)	(0.005)

Notes: Each cell in this table reports a coefficient and standard error (in parentheses) from a separate linear regression. Specifications in column 1 test whether infant characteristics are changing differentially at adopting NICUs relative to non-adopting NICUs, in a way that is correlated with the timing of probiotic adoption. Specifications in column 2 test whether individual infants who receive probiotics are systematically different from infants within the same NICU who do not receive probiotics. Regressions control for hospital and year fixed effects. Sample size: 307,905 infants. ***indicates statistical significance at the 1% level.

© 2023 Agha L et al. *JAMA Health Forum*.

eTable 4. Alternative regression specifications, varying sample and definition of probiotic adoption

	Dependent variable:		
	**Necrotizing enterocolitis**	**Sepsis**	**In-hospital mortality**
Independent variable:	*A. Continuous adoption variable*		
Proportion of VLBW infants given probiotics	0.743***	1.061	1.048
	(0.060)	(0.065)	(0.078)
	B. Sample restricted to NICUs with 8 years of data		
Proportion of VLBW infants given probiotics	0.732***	1.121	1.064
	(0.065)	(0.075)	(0.086)
	C. Sample excluding NICUs that have already adopted probiotics by 2012		
Proportion of VLBW infants given probiotics	0.745***	1.063	1.046
	(0.062)	(0.068)	(0.080)
	D. Binary adoption variable with 20% threshold		
Indicator for Post-Adoption NICU (≥20% probiotic use)	0.805***	1.064	0.987
	(0.051)	(0.053)	(0.059)
	E. Binary adoption variable with 10% threshold		
Indicator for Post-Adoption NICU (≥10% probiotic use)	0.822***	1.032	0.962
	(0.050)	(0.050)	(0.056)

Notes: This table reports results (expressed as odds ratios) and standard errors from 15 separate logit regressions. The outcome variable of the logit regression listed in the column headers (NEC, sepsis, or mortality). The independent variable of interest in Panels A, B, and C is the rate of probiotic use among other VLBW infants in the same NICU-year. For Panels D and E the independent variable of interest is a binary variable indicating that the hospital has used probiotics on at least 20% (Panel D) or at least 10% (Panel E) of VLBW infants in the current year or any prior year. All specifications control for calendar year fixed effects, hospital fixed effects, and neonate characteristics (birth weight, gestational age, SGA, race, sex, multiple, location of birth, 1-minute APGAR score, and major birth defect). Sample size: 307,905 infants. ***indicates statistical significance at the 1% level

© 2023 Agha L et al. *JAMA Health Forum*.

eTable 5. Vermont Oxford Network Members

Hospital	***City***	***State***
Ascension St. Vincent's Birmingham	Birmingham	Alabama
Brookwood Medical Center	Birmingham	Alabama
University of Alabama at Birmingham	Birmingham	Alabama
Huntsville Hospital	Huntsville	Alabama
USA Children's and Women's Hospital	Mobile	Alabama
Baptist Medical Center East	Montgomery	Alabama
Baptist Medical Center South	Montgomery	Alabama
Children's Hospital at Providence, Alaska, The	Anchorage	Alaska
Flagstaff Medical Center	Flagstaff	Arizona
Abrazo Arrowhead Campus	Glendale	Arizona
Banner Children's at Thunderbird	Glendale	Arizona
Banner Children's at Desert	Mesa	Arizona
Arizona Children's Cent Maricopa Integrated Hlth.	Phoenix	Arizona
Banner Estrella Medical Center	Phoenix	Arizona
Banner University Medical Center Phoenix	Phoenix	Arizona
Phoenix Children's Hospital	Phoenix	Arizona
St. Joseph's Hospital and Medical Center	Phoenix	Arizona
HonorHealth Scottsdale Shea Medical Center	Scottsdale	Arizona
Banner University Medical Center - Tucson	Tucson	Arizona
Washington Regional Medical Center	Fayetteville	Arkansas
Mercy Hospital Fort Smith	Fort Smith	Arkansas
Willow Creek Women's Hospital - NICU	Johnson	Arkansas
St. Bernards Medical Center	Jonesboro	Arkansas
Arkansas Children's Hospital	Little Rock	Arkansas
University of Arkansas for Medical Sciences	Little Rock	Arkansas
Mercy Hospital Northwest Arkansas	Rogers	Arkansas
Anaheim Regional Medical Center	Anaheim	California
KFH Orange County - Anaheim	Anaheim	California
St. Mary Medical Center - Apple Valley	Apple Valley	California
Methodist Hospital of Southern California	Arcadia	California
Adventist Health Bakersfield	Bakersfield	California
Bakersfield Memorial Hospital	Bakersfield	California
Kern Medical Center	Bakersfield	California
Mercy Southwest Hospital	Bakersfield	California
KFH Baldwin Park	Baldwin Park	California
Alta Bates Summit Medical Center	Berkeley	California
Providence St. Joseph Medical Center	Burbank	California
Mercy San Juan Medical Center	Carmichael	California

Hospital	***City***	***State***
Rady Children's at Scripps Mercy Chula Vista	Chula Vista	California
Scripps Mercy Hospital Chula Vista	Chula Vista	California
Sharp Chula Vista Medical Center	Chula Vista	California
Clovis Community Medical Center	Clovis	California
Arrowhead Regional Medical Center	Colton	California
Downey Regional Medical Center	Downey	California
KFH Downey	Downey	California
El Centro Regional Medical Center	El Centro	California
Rady Children's at Scripps Encinitas Memorial Hosp	Encinitas	California
Rady Children's at Palomar Medical Center Escondid	Escondido	California
Northbay Medical Center	Fairfield	California
KFH Fontana	Fontana	California
Fountain Valley Regional Hospital & Medical Center	Fountain Valley	California
LPCH Special Care Nursery at Washington Hospital	Fremont	California
Washington Hospital - Fremont	Fremont	California
San Joaquin General Hospital (SJGH)	French Camp	California
Community Regional Med. Center - Fresno	Fresno	California
Valley Children's Hospital at St. Agnes Medical Ce	Fresno	California
St. Jude Medical Center	Fullerton	California
Adventist Health - Glendale	Glendale	California
Glendale Memorial Hospital and Health Center	Glendale	California
MarinHealth Medical Center	Greenbrae	California
KFH South Bay	Harbor City	California
Centinela Hospital Medical Center	Inglewood	California
KFH Orange County - Irvine	Irvine	California
Rady Children's at Scripps La Jolla	La Jolla	California
UCSD Health La Jolla - Jacobs Medical Center	La Jolla	California
Grossmont Hospital, Women's Health Center	La Mesa	California
Memorialcare Saddleback Medical Center	Laguna Hills	California
Antelope Valley Hospital	Lancaster	California
Loma Linda University Children's Hospital	Loma Linda	California
Memorialcare Miller Children's & Women's Hospita	Long Beach	California
St. Mary Medical Center	Long Beach	California
Adventist Health - White Memorial	Los Angeles	California
California Hospital Medical Center - Los Angeles	Los Angeles	California
Cedars-Sinai Medical Center (CSMC)	Los Angeles	California
Children's Hospital Los Angeles (CHLA)	Los Angeles	California
Hollywood Presbyterian Medical Center	Los Angeles	California
KFH Los Angeles	Los Angeles	California
KFH West Los Angeles	Los Angeles	California

© 2023 Agha L et al. *JAMA Health Forum*.

Hospital	***City***	***State***
LAC/USC Medical Center	Los Angeles	California
PIH Health Good Samaritan Hospital	Los Angeles	California
UCLA Mattel Children's Hospital	Los Angeles	California
St. Francis Medical Center	Lynwood	California
Valley Children's Hospital	Madera	California
Providence Holy Cross Medical Center	Mission Hills	California
CHOC Children's at Mission Hospital	Mission Viejo	California
Doctors Medical Center of Modesto	Modesto	California
KFH Modesto	Modesto	California
Memorial Medical Center - Modesto	Modesto	California
Garfield Medical Center	Monterey Park	California
Riverside University Health System Medical Center	Moreno Valley	California
El Camino Hospital	Mountain View	California
Rady Children's at Rancho Springs Medical Center	Murrieta	California
Hoag Memorial Hospital, Presbyterian	Newport Beach	California
Northridge Hospital Medical Center	Northridge	California
KFH Oakland	Oakland	California
UCSF Benioff Children's Hospital - Oakland	Oakland	California
Tri-City Medical Center	Oceanside	California
KFH Ontario Campus	Ontario	California
CHOC Children's Hospital	Orange	California
UC Irvine Medical Center	Orange	California
St. John's Regional Medical Center	Oxnard	California
Desert Regional Medical Center	Palm Springs	California
Lucile Packard Children's Hospital Stanford	Palo Alto	California
KFH Panorama City	Panorama City	California
Huntington Memorial Hospital	Pasadena	California
Pomona Valley Hospital Medical Center	Pomona	California
Mercy Medical Center - Redding	Redding	California
Redlands Community Hospital	Redlands	California
LPCH Special Care Nursery at Sequoia Hospital	Redwood City	California
AHMC Parkview Community Medical Center	Riverside	California
KFH Riverside	Riverside	California
Riverside Community Hospital	Riverside	California
KFH Roseville	Roseville	California
Sutter Roseville Medical Center	Roseville	California
Anderson Lucchetti Women's and Children's Center	Sacramento	California
Methodist Hospital of Sacramento	Sacramento	California
UC Davis Medical Center	Sacramento	California

© 2023 Agha L et al. *JAMA Health Forum*.

Hospital	***City***	***State***
Natividad Medical Center	Salinas	California
Salinas Valley Memorial Hospital	Salinas	California
Community Hospital of San Bernardino	San Bernardino	California
St. Bernardine Medical Center	San Bernardino	California
KFH San Diego	San Diego	California
Rady Children's Hospital San Diego (RCHSD)	San Diego	California
Rady Children's at Scripps Mercy Hospital San Diego	San Diego	California
Scripps Mercy Hospital San Diego	San Diego	California
Sharp Mary Birch Hospital for Women and Newborns	San Diego	California
UCSD Medical Center - Hillcrest	San Diego	California
California Pacific Medical Center Van Ness Campus	San Francisco	California
KFH San Francisco	San Francisco	California
UCSF Benioff Children's Hospital in San Francisco	San Francisco	California
Zuckerberg San Francisco General Hospital and Trau	San Francisco	California
San Gabriel Valley Medical Center	San Gabriel	California
Good Samaritan Hospital, San Jose	San Jose	California
O'Connor Hospital - San Jose	San Jose	California
Regional Medical Center of San Jose	San Jose	California
Santa Clara Valley Medical Center (SCVMC)	San Jose	California
KFH San Leandro	San Leandro	California
Sierra Vista Regional Medical Center	San Luis Obispo	California
Orange County Global Medical Center	Santa Ana	California
Santa Barbara Cottage Hospital	Santa Barbara	California
KFH Santa Clara	Santa Clara	California
Dignity Health Dominican Hospital	Santa Cruz	California
Marian Regional Medical Center	Santa Maria	California
Providence St. John's Health Center	Santa Monica	California
Santa Monica - UCLA Medical Center & Orthopaedic H	Santa Monica	California
Santa Rosa Memorial Hospital	Santa Rosa	California
Sutter Santa Rosa Regional Hospital	Santa Rosa	California
Dameron Hospital Association	Stockton	California
St. Joseph's Medical Center - Stockton	Stockton	California
LAC/Olive View - UCLA Medical Center	Sylmar	California
Providence Cedars-Sinai Tarzana Medical Center	Tarzana	California
Los Robles Regional Hospital & Medical Center	Thousand Oaks	California
LAC/HARBOR - UCLA MEDICAL CENTER	Torrance	California
Providence Little Company of Mary Medical Center	Torrance	California
Torrance Memorial Medical Center	Torrance	California
San Antonio Regional Hospital	Upland	California
Henry Mayo Newhall Memorial Hospital	Valencia	California

© 2023 Agha L et al. *JAMA Health Forum*.

Hospital	***City***	***State***
Valley Presbyterian Hospital	Van Nuys	California
Community Memorial Hospital of Ventura	Ventura	California
Ventura County Medical Center (VCMC)	Ventura	California
Kaweah Delta Healthcare District	Visalia	California
John Muir Health, Walnut Creek Medical Center	Walnut Creek	California
KFH Walnut Creek	Walnut Creek	California
Watsonville Community Hospital	Watsonville	California
Emanate Health - Queen of the Valley Hospital	West Covina	California
Presbyterian Intercommunity Hospital (PIH Health)	Whittier	California
KFH Woodland Hills	Woodland Hills	California
Children's Hospital Colorado	Aurora	Colorado
Medical Center of Aurora	Aurora	Colorado
UCHSC	Aurora	Colorado
St. Francis Medical Center	Colorado Springs	Colorado
Denver Health Medical Center	Denver	Colorado
Rocky Mountain Hospital for Children at P/SL	Denver	Colorado
Rose Medical Center	Denver	Colorado
Saint Joseph Hospital	Denver	Colorado
Swedish Medical Center	Englewood	Colorado
Poudre Valley Hospital	Fort Collins	Colorado
St. Mary's Hospital and Medical Center	Grand Junction	Colorado
Banner Health Northern Colorado Medical Center	Greeley	Colorado
Good Samaritan Medical Center	Lafayette	Colorado
Sky Ridge Medical Center	Lone Tree	Colorado
Medical Center of the Rockies	Loveland	Colorado
Yale-New Haven Children's at Bridgeport Hospital	Bridgeport	Connecticut
Danbury Hospital	Danbury	Connecticut
Connecticut Children's NICU at UCONN Health Center	Farmington	Connecticut
Greenwich Hospital	Greenwich	Connecticut
Connecticut Children's Medical Center	Hartford	Connecticut
St. Francis Hospital	Hartford	Connecticut
Hospital of Central Connecticut, The	New Britain	Connecticut
Yale-New Haven Children's Hospital	New Haven	Connecticut
Norwalk Hospital	Norwalk	Connecticut
Stamford Hospital	Stamford	Connecticut
ChristianaCare	Newark	Delaware
MedStar Georgetown University Hospital	Washington	District Of Columbia
Washington Hospital Center	Washington	District Of Columbia
Brandon Regional Hospital	Brandon	Florida

© 2023 Agha L et al. *JAMA Health Forum*.

Hospital	***City***	***State***
Halifax Medical Center	Daytona Beach	Florida
Broward Health Medical Center/Salah Foundation Chi	Fort Lauderdale	Florida
Golisano Children's Hospital of Southwest Florida	Fort Myers	Florida
Lawnwood Regional Medical Center & Heart Institute	Fort Pierce	Florida
North Florida Regional Medical Center, Inc.	Gainesville	Florida
UF Shands Hospital Gainesville	Gainesville	Florida
Joe DiMaggio Children's Hospital	Hollywood	Florida
UF Health Jacksonville	Jacksonville	Florida
Wolfson Children's Hospital	Jacksonville	Florida
Northwest Medical Center	Margate	Florida
Baptist Children's Hospital	Miami	Florida
Jackson Memorial Hospital	Miami	Florida
Kendall Regional Medical Center	Miami	Florida
Nicklaus Children's Hospital	Miami	Florida
Jackson North Medical Center	North Miami Beach	Florida
AdventHealth for Children	Orlando	Florida
Nemours Children's Hospital	Orlando	Florida
Winnie Palmer Hospital for Women and Babies	Orlando	Florida
Gulf Coast Regional Medical Center	Panama City	Florida
Studer Family Children's Hospital at Ascension Sac	Pensacola	Florida
Plantation General Hospital	Plantation	Florida
Bayfront Health Port Charlotte	Port Charlotte	Florida
Johns Hopkins All Children's Hospital	Saint Petersburg	Florida
All Children's at Sarasota Memorial	Sarasota	Florida
Tallahassee Memorial Hospital	Tallahassee	Florida
St. Joseph's Children's Hospital	Tampa	Florida
Tampa General Hospital	Tampa	Florida
Women's Center - Advent Health Tampa	Tampa	Florida
Wellington Regional Medical Center	Wellington	Florida
St. Mary's Hospital	West Palm Beach	Florida
Phoebe Putney Memorial Hospital	Albany	Georgia
Piedmont Athens Regional	Athens	Georgia
Emory University Hospital, Midtown	Atlanta	Georgia
Grady Memorial Hospital	Atlanta	Georgia
Northside Hospital	Atlanta	Georgia
Piedmont Hospital	Atlanta	Georgia
Augusta University Health System	Augusta	Georgia
Wellstar Cobb Hospital	Austell	Georgia
Medical Center at Columbus Regional, The	Columbus	Georgia

© 2023 Agha L et al. *JAMA Health Forum*.

Hospital	***City***	***State***
Piedmont Rockdale Hospital	Conyers	Georgia
Northside Hospital Forsyth	Cumming	Georgia
Hamilton Medical Center	Dalton	Georgia
WellStar Atlanta Medical Center South	East Point	Georgia
Piedmont Fayette Hospital	Fayetteville	Georgia
Northeast Georgia Medical Center	Gainesville	Georgia
Gwinnett Hospital System	Lawrenceville	Georgia
Coliseum Medical Center	Macon	Georgia
Wellstar Kennestone Hospital	Marietta	Georgia
Southern Regional Medical Center	Riverdale	Georgia
Floyd Medical Center	Rome	Georgia
Memorial Health Savannah	Savannah	Georgia
St. Joseph's/Candler Health System	Savannah	Georgia
Eastside Medical Center	Snellville	Georgia
Piedmont Henry Hospital	Stockbridge	Georgia
Kaiser Permanente Moanalua Medical Center	Honolulu	Hawaii
Kapiolani Medical Center for Women & Children	Honolulu	Hawaii
St. Luke's Regional Medical Center	Boise	Idaho
Kootenai Health	Coeur d'Alene	Idaho
Eastern Idaho Regional Medical Center (EIRMC)	Idaho Falls	Idaho
St. Luke's Meridian Medical Center	Meridian	Idaho
Portneuf Medical Center	Pocatello	Idaho
St. Luke's Magic Valley	Twin Falls	Idaho
Northwest Community Healthcare	Arlington Heights	Illinois
Rush Copley Medical Center	Aurora	Illinois
Memorial Hospital of Carbondale	Carbondale	Illinois
Advocate Illinois Masonic Medical Center	Chicago	Illinois
John H. Stroger, Jr. Hospital of Cook County	Chicago	Illinois
Mercy Hospital and Medical Center	Chicago	Illinois
Mt. Sinai Hospital Medical Center	Chicago	Illinois
Northwestern Memorial	Chicago	Illinois
Rush University Medical Center	Chicago	Illinois
St. Joseph Hospital Chicago	Chicago	Illinois
University of Chicago	Chicago	Illinois
University of Illinois at Chicago	Chicago	Illinois
Advocate Good Samaritan Hospital	Downers Grove	Illinois
Elmhurst Hospital	Elmhurst	Illinois
Evanston Hospital	Evanston	Illinois
Adventist Hinsdale Hospital	Hinsdale	Illinois

© 2023 Agha L et al. *JAMA Health Forum*.

Hospital	***City***	***State***
St. Alexius Medical Center	Hoffman Estates	Illinois
Amita Health St. Joseph Medical Center	Joliet	Illinois
RMCH at Loyola University Medical Center	Maywood	Illinois
Centegra Hospital-McHenry	McHenry	Illinois
UnityPoint Trinity-Moline	Moline	Illinois
Edward Hospital and Health Services	Naperville	Illinois
Advocate Children's Hospital - Oak Lawn	Oak Lawn	Illinois
Advocate Children's Hospital - Park Ridge	Park Ridge	Illinois
CHOI at OSF St. Francis Medical Center	Peoria	Illinois
UnityPoint Health-Methodist Hospital	Peoria	Illinois
Javon Bea Hospital - Riverside	Rockford	Illinois
SwedishAmerican Hospital	Rockford	Illinois
St. John's Hospital	Springfield	Illinois
Carle Foundation Hospital	Urbana	Illinois
Central DuPage Hospital	Winfield	Illinois
Ascension St. Vincent Carmel	Carmel	Indiana
IU Health North Hospital	Carmel	Indiana
Franciscan Health Crown Point	Crown Point	Indiana
Ascension St. Vincent Evansville	Evansville	Indiana
Dupont Hospital	Fort Wayne	Indiana
Lutheran Hospital of Indiana	Fort Wayne	Indiana
Parkview Women's and Children's Hospital	Fort Wayne	Indiana
St. Joseph Hospital	Fort Wayne	Indiana
Methodist Hospitals Northlake Campus	Gary	Indiana
Ascension St. Vincent Hospital - Women's	Indianapolis	Indiana
Community Hospital North Indianapolis	Indianapolis	Indiana
Franciscan St. Francis Health	Indianapolis	Indiana
Methodist Hospital of Indiana	Indianapolis	Indiana
Riley Hospital for Children at IU Health	Indianapolis	Indiana
Wishard Memorial Hospital	Indianapolis	Indiana
Clark Memorial Health	Jeffersonville	Indiana
Franciscan Health Lafayette	Lafayette	Indiana
Saint Joseph Regional Medical Center	Mishawaka	Indiana
Ball Memorial Hospital	Muncie	Indiana
Community Hospital	Munster	Indiana
Women's Hospital	Newburgh	Indiana
Memorial Hospital	South Bend	Indiana
Union Hospital	Terre Haute	Indiana
UnityPoint Trinity-Bettendorf NSCU	Bettendorf	Iowa

© 2023 Agha L et al. *JAMA Health Forum*.

Hospital	***City***	***State***
Mercy Medical Center Cedar Rapids	Cedar Rapids	Iowa
St. Luke's Hospital	Cedar Rapids	Iowa
Genesis Medical Center	Davenport	Iowa
Blank Children's Hospital	Des Moines	Iowa
Mercy Medical Center	Des Moines	Iowa
University of Iowa Children's Hospital	Iowa City	Iowa
St. Luke's Regional Medical Center	Sioux City	Iowa
Covenant Medical Center	Waterloo	Iowa
University of Kansas Hospital Authority	Kansas City	Kansas
Overland Park Regional Medical Center	Overland Park	Kansas
Shawnee Mission Medical Center	Shawnee Mission	Kansas
Ascension Via Christi Hospital	Wichita	Kansas
Wesley Medical Center	Wichita	Kansas
King's Daughters Medical Center	Ashland	Kentucky
Medical Center NICU Bowling Green, The	Bowling Green	Kentucky
St. Elizabeth Healthcare	Edgewood	Kentucky
Hardin Memorial Hospital	Elizabethtown	Kentucky
Frankfort Regional Medical Center	Frankfort	Kentucky
Deaconess Henderson Hospital	Henderson	Kentucky
Jennie Stuart Medical Center	Hopkinsville	Kentucky
Baptist Health Lexington	Lexington	Kentucky
Kentucky Children's Hospital	Lexington	Kentucky
Women's Hospital at St. Joseph East, The	Lexington	Kentucky
Baptist Health Louisville	Louisville	Kentucky
Norton Children's Hospital	Louisville	Kentucky
Norton Women's and Children's Hospital	Louisville	Kentucky
University of Louisville Hospital	Louisville	Kentucky
Baptist Health Deaconess Madisonville	Madisonville	Kentucky
Owensboro Health Regional Hospital	Owensboro	Kentucky
Baptist Health Paducah	Paducah	Kentucky
Pikeville Medical Center	Pikeville	Kentucky
Christus St. Frances Cabrini Hospital	Alexandria	Louisiana
Rapides Women's and Children's Hospital	Alexandria	Louisiana
Baton Rouge General Medical Ctr - Bluebonnet	Baton Rouge	Louisiana
Ochsner Medical Center Baton Rouge	Baton Rouge	Louisiana
Woman's Hospital	Baton Rouge	Louisiana
Lakeview Regional Medical Center	Covington	Louisiana
St. Tammany Parish Hospital	Covington	Louisiana
Ochsner Medical Center WestBank	Gretna	Louisiana

© 2023 Agha L et al. *JAMA Health Forum*.

Hospital	***City***	***State***
North Oaks Medical Center	Hammond	Louisiana
Terrebonne General Medical Center	Houma	Louisiana
Lafayette General Medical Center	Lafayette	Louisiana
Our Lady of Lourdes Women's & Children's Hospital	Lafayette	Louisiana
Christus Lake Area Hospital	Lake Charles	Louisiana
Lake Charles Memorial Hosp for Women	Lake Charles	Louisiana
West Jefferson Medical Center	Marrero	Louisiana
East Jefferson General Hospital	Metairie	Louisiana
Tulane Lakeside Hospital	Metairie	Louisiana
St. Francis Medical Center	Monroe	Louisiana
Children's Hospital of New Orleans	New Orleans	Louisiana
Medical Center of Louisiana at New Orleans	New Orleans	Louisiana
Ochsner Baptist Medical Center	New Orleans	Louisiana
Ochsner Medical Center - Jefferson Hwy	New Orleans	Louisiana
Touro Infirmary	New Orleans	Louisiana
CHRISTUS Highland	Shreveport	Louisiana
Ochsner LSU Health St. Mary Medical Center	Shreveport	Louisiana
Ochsner LSU Health St. Mary Medical Center	Shreveport	Louisiana
Willis Knighton South	Shreveport	Louisiana
Slidell Memorial Hospital	Slidell	Louisiana
Eastern Maine Medical Center	Bangor	Maine
Barbara Bush Children's at Maine Medical	Portland	Maine
Anne Arundel Medical Center	Annapolis	Maryland
Ascension Saint Agnes Hospital	Baltimore	Maryland
Franklin Square Hospital Center	Baltimore	Maryland
Greater Baltimore Medical Center	Baltimore	Maryland
JHBMC Hopkins Bayview Medical Ctr	Baltimore	Maryland
Johns Hopkins Hospital	Baltimore	Maryland
Mercy Medical Center - Baltimore	Baltimore	Maryland
Sinai Hospital of Baltimore	Baltimore	Maryland
Univ of Maryland Medical Center	Baltimore	Maryland
Walter Reed Nat'l Mil Med Center NICU	Bethesda	Maryland
University of Maryland Capital Region Medical	Cheverly	Maryland
Howard County General Hospital	Columbia	Maryland
Frederick Memorial Hospital	Frederick	Maryland
Shady Grove Adventist Hospital	Rockville	Maryland
Peninsula Regional Medical Center	Salisbury	Maryland
Holy Cross Hospital	Silver Spring	Maryland
Univ of Maryland St. Joseph Medical Ctr.	Towson	Maryland

© 2023 Agha L et al. *JAMA Health Forum*.

Hospital	***City***	***State***
Beth Israel Deaconess Medical Center	Boston	Massachusetts
Boston Medical Center	Boston	Massachusetts
Brigham and Women's Hospital	Boston	Massachusetts
Massachusetts General Hospital for Children	Boston	Massachusetts
Tufts Medical Center	Boston	Massachusetts
Steward St. Elizabeth's Medical Center	Brighton	Massachusetts
South Shore Hospital	South Weymouth	Massachusetts
Baystate Medical Center	Springfield	Massachusetts
UMass Memorial Healthcare	Worcester	Massachusetts
St. Joseph Mercy Hospital	Ann Arbor	Michigan
U. of MI, CS Mott Children's, Brandon NICU	Ann Arbor	Michigan
Beaumont Hospital - Dearborn	Dearborn	Michigan
Ascension St. John Hospital	Detroit	Michigan
Children's Hospital of Michigan	Detroit	Michigan
DMC Sinai-Grace Hospital	Detroit	Michigan
Henry Ford Hospital	Detroit	Michigan
Hutzel Women's Hospital	Detroit	Michigan
Hurley Medical Center	Flint	Michigan
Helen DeVos Children's Hospital	Grand Rapids	Michigan
Mercy Health Saint Mary's	Grand Rapids	Michigan
Henry Ford Allegiance Health	Jackson	Michigan
Children's Hospital at Bronson	Kalamazoo	Michigan
Sparrow Hospital	Lansing	Michigan
UPHS-Marquette	Marquette	Michigan
St. Joseph Mercy Oakland	Pontiac	Michigan
William Beaumont Hospital	Royal Oak	Michigan
Covenant Healthcare	Saginaw	Michigan
Ascension Providence Hospital Southfield Campus	Southfield	Michigan
Munson Medical Center	Traverse City	Michigan
Essentia Health - St. Mary's Children's Hospital	Duluth	Minnesota
Maple Grove Hospital	Maple Grove	Minnesota
Children's Minnesota - Minneapolis	Minneapolis	Minnesota
Hennepin County Medical Center	Minneapolis	Minnesota
University of MN Masonic Children's Hospital	Minneapolis	Minnesota
North Memorial Medical Center	Robbinsdale	Minnesota
Mayo Foundation	Rochester	Minnesota
St. Cloud Hospital	Saint Cloud	Minnesota
Children's Minnesota-St. Paul	Saint Paul	Minnesota
Merit Health River Oaks	Flowood	Mississippi

© 2023 Agha L et al. *JAMA Health Forum*.

Hospital	*City*	*State*
Merit Health Woman's Hospital	Flowood	Mississippi
Delta Regional Medical Center	Greenville	Mississippi
Memorial Hospital at Gulfport	Gulfport	Mississippi
Forrest General Hospital	Hattiesburg	Mississippi
Merit Health Wesley	Hattiesburg	Mississippi
Children's Hospital at U. of MS Health Care	Jackson	Mississippi
Merit Health Central	Jackson	Mississippi
Mississippi Baptist Health Systems	Jackson	Mississippi
St. Dominic, Jackson Memorial Hospital	Jackson	Mississippi
Anderson Regional Medical Center	Meridian	Mississippi
North Mississippi Medical Center	Tupelo	Mississippi
SoutheastHEALTH	Cape Girardeau	Missouri
St. Francis Medical Center	Cape Girardeau	Missouri
Women's & Children's Hosp, U. of MO	Columbia	Missouri
Centerpoint Medical Center	Independence	Missouri
Freeman Hospital and Health System	Joplin	Missouri
Mercy Hospital Joplin	Joplin	Missouri
Children's Mercy Kansas City	Kansas City	Missouri
Research Medical Center	Kansas City	Missouri
St. Luke's Hospital	Kansas City	Missouri
Truman Medical Center	Kansas City	Missouri
Mercy Children's Hospital , St. Louis	Saint Louis	Missouri
SSM Health Cardinal Glennon Children's Hospital	Saint Louis	Missouri
St. Louis Children's Hospital	Saint Louis	Missouri
Cox Health - Neonatology	Springfield	Missouri
Mercy Kids Springfield	Springfield	Missouri
Billings Clinic	Billings	Montana
St. Vincent Hospital & Health Center	Billings	Montana
Benefis Healthcare	Great Falls	Montana
Kalispell Regional Medical Center	Kalispell	Montana
Community Medical Center	Missoula	Montana
Good Samaritan Hospital Kearney	Kearney	Nebraska
Bryan Medical Center	Lincoln	Nebraska
CHI Health St. Elizabeth	Lincoln	Nebraska
CHI Health Bergan Mercy Medical Center	Omaha	Nebraska
Children's Hospital and Medical Center	Omaha	Nebraska
Creighton University Medical Center	Omaha	Nebraska
Methodist Women's Hospital	Omaha	Nebraska
Nebraska Medical Center	Omaha	Nebraska

© 2023 Agha L et al. *JAMA Health Forum*.

Hospital	***City***	***State***
St. Rose Dominican Hospital Siena Campus	Henderson	Nevada
Children's Hospital of Nevada at UMC	Las Vegas	Nevada
MountainView Hospital	Las Vegas	Nevada
Sunrise Hospital and Medical Center	Las Vegas	Nevada
Dartmouth Hitchcock Medical Center	Lebanon	New Hampshire
Elliot Hospital	Manchester	New Hampshire
Children's at Cooper University Medical Center	Camden	New Jersey
Our Lady of Lourdes Medical Center	Camden	New Jersey
JFK Medical Center	Edison	New Jersey
Englewood Hospital and Medical Center	Englewood	New Jersey
CentraState Medical Center	Freehold	New Jersey
Hackensack University Medical Center	Hackensack	New Jersey
RWJUH Hamilton	Hamilton	New Jersey
Jersey City Medical Center	Jersey City	New Jersey
Monmouth Medical Center Southern Campus	Lakewood	New Jersey
Saint Barnabas Medical Center	Livingston	New Jersey
Monmouth Medical Center	Long Branch	New Jersey
Mountainside Hospital	Montclair	New Jersey
Goryeb Children's Hospital	Morristown	New Jersey
Jersey Shore University Medical Center	Neptune	New Jersey
Robert Wood Johnson University Hospital	New Brunswick	New Jersey
St. Peter's Medical Center	New Brunswick	New Jersey
Children's Hospital of New Jersey at NBIMC	Newark	New Jersey
University Hospital Rutgers-NJMS	Newark	New Jersey
St. Joseph Hospital and Medical Center	Paterson	New Jersey
Capital Health Medical Center-Hopewell	Pennington	New Jersey
Penn Medicine Princeton Medical Center	Plainsboro	New Jersey
Atlanticare Regional Medical Center - NICU	Pomona	New Jersey
Shore Medical Center	Somers Point	New Jersey
Overlook Medical Center	Summit	New Jersey
Community Medical Center Toms River	Toms River	New Jersey
Inspira Health Network	Vineland	New Jersey
Virtua Hospital Systems	Voorhees	New Jersey
Hackensack UMC @ Pascack Valley	Westwood	New Jersey
Children's Medical Center at Presbyterian Hospital	Albuquerque	New Mexico
Lovelace Women's Hospital	Albuquerque	New Mexico
University of New Mexico Health Sciences Center	Albuquerque	New Mexico
Albany Medical Center	Albany	New York
St. Peter's Hospital	Albany	New York

© 2023 Agha L et al. *JAMA Health Forum*.

Hospital	***City***	***State***
Montefiore Medical Center-Wakefield Division	Bronx	New York
NYC Health + Hospitals - Jacobi Medical Center	Bronx	New York
Weiler Hospital Montefiore	Bronx	New York
Brookdale Hospital Medical Center	Brooklyn	New York
Brooklyn Hospital Center, The	Brooklyn	New York
Maimonides Medical Center	Brooklyn	New York
NYC Health + Hospitals - Kings County	Brooklyn	New York
NYU Langone Hospital-Brooklyn	Brooklyn	New York
NYU Lutheran Medical Center	Brooklyn	New York
University Hospital of Brooklyn	Brooklyn	New York
Woodhull Medical Center	Brooklyn	New York
Sisters of Charity Hospital	Buffalo	New York
NYC Health + Hospitals - Elmhurst	Elmhurst	New York
Arnot Ogden Medical Center	Elmira	New York
NYC Health + Hospitals - Queens Hospital Center	Jamaica	New York
North Shore University Hospital	Manhasset	New York
Orange Regional Medical Center	Middletown	New York
NYU Winthrop	Mineola	New York
Northern Westchester Hospital	Mount Kisco	New York
Cohen Children's Medical Center of New York	New Hyde Park	New York
Columbia University Medical Center	New York	New York
Lenox Hill Hospital	New York	New York
Mt. Sinai Kravis Children's Hospital, The	New York	New York
NYC Health + Hospitals - Bellevue	New York	New York
Tisch Hospital, NYU Medical Center	New York	New York
Weill Cornell Medical Center	New York	New York
Vassar Brothers Hospital	Poughkeepsie	New York
Golisano Children's Hospital at Strong	Rochester	New York
Richmond University Medical Center	Staten Island	New York
Staten Island University Hospital	Staten Island	New York
Stony Brook University Medical Center	Stony Brook	New York
Crouse Health Baker Regional NICU	Syracuse	New York
St. Joseph's Health Center	Syracuse	New York
Good Samaritan Hospital Medical Center	West Islip	New York
Mission Children's Hospital	Asheville	North Carolina
North Carolina Children's Hospital	Chapel Hill	North Carolina
Carolinas Medical Center	Charlotte	North Carolina
Novant Health Presbyterian Medical Center	Charlotte	North Carolina
Jeff Gordon Children's Hospital at CMC	Concord	North Carolina

© 2023 Agha L et al. *JAMA Health Forum*.

Hospital	***City***	***State***
Duke University	Durham	North Carolina
Cape Fear Valley Medical Center	Fayetteville	North Carolina
CaroMont Regional Medical Center	Gastonia	North Carolina
Cone Health Women & Children's Center at Moses Con	Greensboro	North Carolina
Vidant Medical Center	Greenville	North Carolina
Catawba Valley Medical Center	Hickory	North Carolina
Frye Regional Medical Center	Hickory	North Carolina
Onslow Memorial Hospital	Jacksonville	North Carolina
FirstHealth Moore Regional Hospital	Pinehurst	North Carolina
Rex Hospital	Raleigh	North Carolina
WakeMedical Center	Raleigh	North Carolina
Novant Health New Hanover Regional Medical Center	Wilmington	North Carolina
Brenner Children's Hospital at WFUBMC	Winston-Salem	North Carolina
Forsyth Memorial Hospital	Winston-Salem	North Carolina
Sanford Bismarck Medical Center	Bismarck	North Dakota
Essentia Health	Fargo	North Dakota
Sanford Medical Center Fargo	Fargo	North Dakota
Altru Health System	Grand Forks	North Dakota
Trinity Hospital	Minot	North Dakota
Akron Children's Hospital	Akron	Ohio
Akron Children's Special Care Nursery at Summa	Akron	Ohio
Akron Children's NICU at St. Elizabeth - Boardman	Boardman	Ohio
Akron Children's NICU at Aultman	Canton	Ohio
Children's Hospital Medical Center Cincinnati	Cincinnati	Ohio
Good Samaritan Hospital	Cincinnati	Ohio
University Hospital, Cincinnati	Cincinnati	Ohio
Cleveland Clinic Foundation, The	Cleveland	Ohio
Fairview Hospital	Cleveland	Ohio
MetroHealth Medical Center	Cleveland	Ohio
Rainbow Babies & Children's Hospital	Cleveland	Ohio
Mount Carmel East	Columbus	Ohio
NCH Main Campus	Columbus	Ohio
NCH NICU @Ohio State Medical Ctr	Columbus	Ohio
NCH at Doctor's Hospital West	Columbus	Ohio
NCH at Grant Medical Center	Columbus	Ohio
NCH at Riverside Methodist Hospital	Columbus	Ohio
Dayton Children's Hospital	Dayton	Ohio
Miami Valley Hospital	Dayton	Ohio

© 2023 Agha L et al. *JAMA Health Forum*.

Hospital	***City***	***State***
Southview Medical Center	Dayton	Ohio
NCH at Dublin Methodist Hospital	Dublin	Ohio
Mount Carmel Grove City	Grove City	Ohio
Kettering Medical Center	Kettering	Ohio
St. Rita's Medical Center	Lima	Ohio
CCF Children's - Hillcrest NICU	Mayfield Heights	Ohio
Mercy Children's Hospital	Toledo	Ohio
Russell J. Ebeid Children's Hospital	Toledo	Ohio
NCH NICU at Mount Carmel St. Ann's Hospital	Westerville	Ohio
Mercy Health Center	Oklahoma City	Oklahoma
Mercy Hospital Oklahoma City	Oklahoma City	Oklahoma
O.U. Health Sciences Center	Oklahoma City	Oklahoma
Ascension St John Medical Center	Tulsa	Oklahoma
Henry Zarrow Neonatal Intensive Care Unit	Tulsa	Oklahoma
OSU Medical Center	Tulsa	Oklahoma
Peggy V. Helmerich Women's Center	Tulsa	Oklahoma
St. Charles Health Care	Bend	Oregon
Kaiser Sunnyside Medical Center	Clackamas	Oregon
Rogue Regional Medical Center	Medford	Oregon
Oregon Health and Science University	Portland	Oregon
Providence Portland Medical Center	Portland	Oregon
Providence St. Vincent Medical Center	Portland	Oregon
Randall Children's Hospital at Legacy Emanuel	Portland	Oregon
Salem Hospital	Salem	Oregon
PeaceHealth Riverbend	Springfield	Oregon
Abington Memorial Hospital	Abington	Pennsylvania
Lehigh Valley Health Network	Allentown	Pennsylvania
St. Luke's Allentown Campus	Allentown	Pennsylvania
St. Luke's University Hospital	Bethlehem	Pennsylvania
Bryn Mawr Hospital	Bryn Mawr	Pennsylvania
Holy Spirit Hospital	Camp Hill	Pennsylvania
Geisinger Medical Center	Danville	Pennsylvania
Doylestown Hospital	Doylestown	Pennsylvania
Penn Highlands DuBois	DuBois	Pennsylvania
Einstein Medical Center Montgomery	East Norriton	Pennsylvania
Ephrata Community Hospital	Ephrata	Pennsylvania
Hamot Medical Center	Erie	Pennsylvania
St. Vincent Health Center	Erie	Pennsylvania
Pinnacle Health Hospitals	Harrisburg	Pennsylvania

© 2023 Agha L et al. *JAMA Health Forum*.

Hospital	*City*	*State*
Penn State Children's Hospital	Hershey	Pennsylvania
Conemaugh Memorial Medical Center	Johnstown	Pennsylvania
Lancaster General Health-Women & Babies Hospital	Lancaster	Pennsylvania
St. Mary Medical Center	Langhorne	Pennsylvania
Holy Redeemer Hospital and Medical Center	Meadowbrook	Pennsylvania
Riddle Hospital	Media	Pennsylvania
Children's Hospital of Philadelphia Newborn Center	Philadelphia	Pennsylvania
Einstein Medical Center Philadelphia	Philadelphia	Pennsylvania
Hahnemann University Hospital	Philadelphia	Pennsylvania
Hospital of the University of Pennsylvania	Philadelphia	Pennsylvania
Pennsylvania Hospital	Philadelphia	Pennsylvania
Temple University Hospital	Philadelphia	Pennsylvania
Thomas Jefferson University Hospital	Philadelphia	Pennsylvania
Magee-Womens Hospital of UPMC	Pittsburgh	Pennsylvania
Western Pennsylvania Hospital	Pittsburgh	Pennsylvania
Reading Hospital-Tower Health	Reading	Pennsylvania
Moses Taylor Hospital	Scranton	Pennsylvania
Crozer Chester Medical Center	Upland	Pennsylvania
CHOP Newborn Care at Chester County Hospital	West Chester	Pennsylvania
Geisinger Wyoming Valley Medical Center	Wilkes-Barre	Pennsylvania
Lankenau Medical Center	Wynnewood	Pennsylvania
WellSpan York Hospital	York	Pennsylvania
Women & Infants Hospital	Providence	Rhode Island
Shawn Jenkins Children's Hospital	Charleston	South Carolina
Trident Medical Center	Charleston	South Carolina
Palmetto Health Richland	Columbia	South Carolina
Prisma Health Baptist	Columbia	South Carolina
McLeod Regional Medical Center	Florence	South Carolina
Children's Hospital of Greenville	Greenville	South Carolina
St. Francis Eastside	Greenville	South Carolina
Piedmont Medical Center	Rock Hill	South Carolina
Spartanburg Regional Healthcare System	Spartanburg	South Carolina
Summerville Medical Center	Summerville	South Carolina
Monument Health Rapid City Hospital	Rapid City	South Dakota
Avera McKennan	Sioux Falls	South Dakota
Boekelheide NICU at Sanford Health	Sioux Falls	South Dakota
Children's Hospital at Erlanger	Chattanooga	Tennessee
Parkridge East Hospital	Chattanooga	Tennessee
Maury Regional Medical Center	Columbia	Tennessee

© 2023 Agha L et al. *JAMA Health Forum*.

Hospital	***City***	***State***
TriStar Summit Medical Center	Hermitage	Tennessee
Jackson Madison County General Hospital	Jackson	Tennessee
Niswonger Children's Hospital	Johnson City	Tennessee
Wellmont Holston Valley Medical Center	Kingsport	Tennessee
East Tennessee Children's Hospital	Knoxville	Tennessee
St. Mary's Health System Inc.	Knoxville	Tennessee
University of Tennessee Medical Center	Knoxville	Tennessee
Baptist Memorial Hospital for Women	Memphis	Tennessee
Le Bonheur Children's Hospital	Memphis	Tennessee
Regional One Health	Memphis	Tennessee
St. Francis Hospital Memphis	Memphis	Tennessee
Children's Hospital at TriStar Centennial, The	Nashville	Tennessee
Monroe Carell Jr. Children's Hospital Vanderbilt	Nashville	Tennessee
Hendrick Medical Center	Abilene	Texas
Hendrick Medical Center South	Abilene	Texas
Baptist St. Anthony's Health System	Amarillo	Texas
Northwest Texas Healthcare System	Amarillo	Texas
Medical City Arlington	Arlington	Texas
Texas Health Arlington Memorial Hospital	Arlington	Texas
Ascension Seton Medical Center Austin	Austin	Texas
Dell Children's Medical Center of Central Texas	Austin	Texas
North Austin Medical Center	Austin	Texas
St. David's Medical Center	Austin	Texas
St. David's South Austin Medical Center	Austin	Texas
CHRISTUS Southeast Texas Hospital	Beaumont	Texas
Valley Baptist Medical Center - Brownsville	Brownsville	Texas
Valley Regional Medical Center TX	Brownsville	Texas
HCA Houston Healthcare Conroe	Conroe	Texas
Christus Spohn Hospital Corpus Christi South	Corpus Christi	Texas
Corpus Christi Medical Center	Corpus Christi	Texas
Driscoll Children's Hospital	Corpus Christi	Texas
Memorial Hermann Cypress Hospital	Cypress	Texas
Baylor Healthcare System	Dallas	Texas
Medical City Dallas	Dallas	Texas
Methodist Dallas Medical Center	Dallas	Texas
Presbyterian Hospital of Dallas	Dallas	Texas
Texas Health Presbyterian Hospital Dallas	Dallas	Texas
University of Texas Southwestern Med. Ctr. Dallas	Dallas	Texas
William P. Clements Jr. University Hospital	Dallas	Texas

© 2023 Agha L et al. *JAMA Health Forum*.

Hospital	***City***	***State***
Texas Health Presbyterian Hospital Denton	Denton	Texas
Women's Hospital At Renaissance	Edinburg	Texas
Del Sol Medical Center	El Paso	Texas
El Paso Children's Hospital	El Paso	Texas
Las Palmas Medical Center	El Paso	Texas
University Medical Center of El Paso	El Paso	Texas
Texas Health Presbyterian Hospital Flower Mound	Flower Mound	Texas
Baylor All Saints Medical Center	Fort Worth	Texas
Cook Children's Medical Center	Fort Worth	Texas
Harris Methodist Fort Worth Hospital	Fort Worth	Texas
John Peter Smith Hospital	Fort Worth	Texas
Medical City Alliance	Fort Worth	Texas
Medical City Frisco	Frisco	Texas
University of Texas Medical Branch	Galveston	Texas
Valley Baptist Medical Center - Harlingen	Harlingen	Texas
Ben Taub General Hospital	Houston	Texas
Children's Memorial Hermann Hospital	Houston	Texas
HCA Houston Healthcare Cypress Fairbanks	Houston	Texas
Houston Methodist West Hospital	Houston	Texas
Lyndon B. Johnson General Hospital	Houston	Texas
Memorial Hermann Greater Heights Hospital	Houston	Texas
Memorial Hermann Memorial City Med Ctr	Houston	Texas
Memorial Hermann Southeast	Houston	Texas
Memorial Hermann Southwest	Houston	Texas
Methodist Willowbrook Hospital	Houston	Texas
St. Joseph Hospital	Houston	Texas
Texas Children's Hospital, Baylor College of Med.	Houston	Texas
Woman's Hospital of Texas, The	Houston	Texas
Memorial Hermann Northeast Hospital	Humble	Texas
Memorial Hermann Katy Hospital	Katy	Texas
HCA Houston Healthcare Kingwood	Kingwood	Texas
Medical City Lewisville	Lewisville	Texas
Christus Good Shepherd Medical Center	Longview	Texas
Longview Regional Medical Center	Longview	Texas
Covenant Children's Hospital	Lubbock	Texas
Methodist Mansfield Medical Center	Mansfield	Texas
Rio Grande Regional Hospital	McAllen	Texas
Medical City of McKinney	McKinney	Texas
HCA Houston Healthcare Southeast	Pasadena	Texas

© 2023 Agha L et al. *JAMA Health Forum*.

Hospital	***City***	***State***
Medical City Plano	Plano	Texas
Texas Health Presbyterian Hospital Plano	Plano	Texas
Methodist Richardson Medical Center	Richardson	Texas
Lake Pointe Medical Center	Rowlett	Texas
Children's Hospital of San Antonio	San Antonio	Texas
Methodist Children's Hospital	San Antonio	Texas
Methodist Stone Oak Hospital (MSOH)	San Antonio	Texas
Metropolitan Methodist Hospital	San Antonio	Texas
North Central Baptist Hospital	San Antonio	Texas
St. Luke's Baptist Hospital	San Antonio	Texas
University Hospital San Antonio	San Antonio	Texas
Houston Methodist Sugar Land Hospital	Sugar Land	Texas
Baylor Scott & White McLane Children's Medical Cen	Temple	Texas
Christus St. Michael Health System	Texarkana	Texas
Houston Methodist The Woodlands Hospital	The Woodlands	Texas
Memorial Hermann The Woodlands	The Woodlands	Texas
St. Luke's The Woodlands Hospital	The Woodlands	Texas
Texas Children's Hospital The Woodlands	The Woodlands	Texas
Christus Trinity Mother Frances Health System	Tyler	Texas
DeTar Hospital North	Victoria	Texas
Baylor Scott & White Medical Center - Hillcrest	Waco	Texas
HCA Houston Healthcare Clear Lake	Webster	Texas
Intermountain Medical Center	Murray	Utah
McKay Dee Hospital Center	Ogden	Utah
Ogden Regional Medical Center	Ogden	Utah
Timpanogos Regional Hospital	Orem	Utah
Utah Valley Regional Medical Center	Provo	Utah
St. George Regional Medical Center	Saint George	Utah
St. Mark's Hospital	Salt Lake City	Utah
University of Utah Health Sciences Center	Salt Lake City	Utah
University of Vermont Children's Hospital	Burlington	Vermont
Inova Alexandria Hospital (NICU)	Alexandria	Virginia
Virginia Hospital Center	Arlington	Virginia
University of Virginia	Charlottesville	Virginia
Inova Fair Oaks Hospital	Fairfax	Virginia
INOVA Children's Hospital	Falls Church	Virginia
Mary Washington Hospital	Fredericksburg	Virginia
Spotsylvania Regional Medical Center	Fredericksburg	Virginia
Inova Loudoun Hospital	Leesburg	Virginia
Centra Health, Virginia Baptist Hospital	Lynchburg	Virginia

© 2023 Agha L et al. *JAMA Health Forum*.

Hospital	*City*	*State*
Children's Hospital of the King's Daughters	Norfolk	Virginia
Reston Hospital Center	Reston	Virginia
Bon Secours St. Mary's Hospital	Richmond	Virginia
CJW Medical Center, Chippenham Campus	Richmond	Virginia
Children's Hosp of Richmond at VCU	Richmond	Virginia
Henrico Doctors' Hospital	Richmond	Virginia
Johnston-Willis Hospital	Richmond	Virginia
Carilion Clinic Children's Hospital	Roanoke	Virginia
Winchester Medical Center	Winchester	Virginia
Sentara Northern Virginia Medical Center	Woodbridge	Virginia
Overlake Hospital Medical Center	Bellevue	Washington
Providence Regional Medical Center Everett	Everett	Washington
Evergreen Health	Kirkland	Washington
UW Medicine - Valley Medical Center	Renton	Washington
Kadlec Regional Medical Center	Richland	Washington
Swedish Med Cen-First Hill Pavilion	Seattle	Washington
University of Washington Medical Center	Seattle	Washington
Deaconess Hospital	Spokane	Washington
Providence Sacred Heart Medical Center & Childrens	Spokane	Washington
MultiCare Health System-Tacoma General Hospital	Tacoma	Washington
St. Joseph Medical Center	Tacoma	Washington
Legacy Salmon Creek Hospital	Vancouver	Washington
PeaceHealth Southwest Medical Center	Vancouver	Washington
Yakima Valley Memorial Hospital	Yakima	Washington
Charleston Area Medical Center	Charleston	West Virginia
Cabell Huntington Hospital, Inc.	Huntington	West Virginia
Berkeley Medical Center	Martinsburg	West Virginia
West Virginia University School of Medicine	Morgantown	West Virginia
Ascension NE Wisconsin - St Elizabeth Campus	Appleton	Wisconsin
Aurora Baycare Medical Center	Green Bay	Wisconsin
St. Vincent Hospital	Green Bay	Wisconsin
Gundersen Lutheran Medical Center	La Crosse	Wisconsin
American Family Children's Hospital	Madison	Wisconsin
Meriter Hospital	Madison	Wisconsin
SSM Health St. Mary's Hospital Madison	Madison	Wisconsin
Marshfield Medical Center - Marshfield	Marshfield	Wisconsin
Ascension Columbia St. Mary's Hospital Milwaukee	Milwaukee	Wisconsin
Ascension SE Wisconsin Hospital - St Joseph Campus	Milwaukee	Wisconsin

© 2023 Agha L et al. *JAMA Health Forum*.

Hospital	***City***	***State***
Aurora Sinai Medical Center	Milwaukee	Wisconsin
Children's Hospital of Wisconsin, Fox Valley	Neenah	Wisconsin
Ascension All Saints Hospital	Racine	Wisconsin
Waukesha Memorial Hospital	Waukesha	Wisconsin
Aspirus Wausau Hospital	Wausau	Wisconsin
Children's Wisconsin	Wauwatosa	Wisconsin
Aurora Women's Pavilion	West Allis	Wisconsin

© 2023 Agha L et al. *JAMA Health Forum*.
