# Supplementary material for: Association of Hospital Adoption of Probiotics With Outcomes Among Neonates With Very Low Birth Weight
Source: JAMA Health Forum. 2023 May 12;4(5):e230960. doi: 10.1001/jamahealthforum.2023.0960 (PMC10182437; doi:10.1001/jamahealthforum.2023.0960)
Supplement: Supplement 2. — Data Sharing Statement [file jamahealthforum-e230960-s002.pdf]

Data Sharing Statement

Agha. Association of Hospital Adoption of Probiotics With Outcomes Among Neonates With Very Low Birth Weight. *JAMA Health Forum*. Published May 12, 2023. doi:10.1001/jamahealthforum.2023.0960

Data
Data available: No

Additional Information
Explanation for why data not available: This research is based on protected patient data collected by the Vermont Oxford Network, a nonprofit collaborative that collects data on neonatal care.
